# Selective gene-expression profiling of migratory tumor cells *in vivo *predicts clinical outcome in breast cancer patients

**DOI:** 10.1186/bcr3344

**Published:** 2012-10-31

**Authors:** Antonia Patsialou, Yarong Wang, Juan Lin, Kathleen Whitney, Sumanta Goswami, Paraic A Kenny, John S Condeelis

**Affiliations:** 1 Department of Anatomy and Structural Biology, Albert Einstein College of Medicine, 1300 Morris Park Ave, Bronx, NY 10461, USA; 2 Department of Epidemiology and Population Health, Division of Biostatistics, Albert Einstein College of Medicine, 1300 Morris Park Ave, Bronx, NY 10461, USA; 3 Department of Pathology, Albert Einstein College of Medicine, 1300 Morris Park Ave, Bronx, NY 10461, USA; 4 Department of Developmental and Molecular Biology, Albert Einstein College of Medicine, 1300 Morris Park Ave, Bronx, NY 10461, USA; 5 Montefiore Medical Center, 1825 Eastchester Road, Bronx, NY 10461, USA; 6 Department of Biology, Yeshiva University, 500 West 185th Street, New York, NY 10033, USA

## Abstract

**Introduction:**

Metastasis of breast cancer is the main cause of death in patients. Previous genome-wide studies have identified gene-expression patterns correlated with cancer patient outcome. However, these were derived mostly from whole tissue without respect to cell heterogeneity. In reality, only a small subpopulation of invasive cells inside the primary tumor is responsible for escaping and initiating dissemination and metastasis. When whole tissue is used for molecular profiling, the expression pattern of these cells is masked by the majority of the noninvasive tumor cells. Therefore, little information is available about the crucial early steps of the metastatic cascade: migration, invasion, and entry of tumor cells into the systemic circulation.

**Methods:**

In the past, we developed an *in vivo *invasion assay that can capture specifically the highly motile tumor cells in the act of migrating inside living tumors. Here, we used this assay in orthotopic xenografts of human MDA-MB-231 breast cancer cells to isolate selectively the migratory cell subpopulation of the primary tumor for gene-expression profiling. In this way, we derived a gene signature specific to breast cancer migration and invasion, which we call the Human Invasion Signature (HIS).

**Results:**

Unsupervised analysis of the HIS shows that the most significant upregulated gene networks in the migratory breast tumor cells include genes regulating embryonic and tissue development, cellular movement, and DNA replication and repair. We confirmed that genes involved in these functions are upregulated in the migratory tumor cells with independent biological repeats. We also demonstrate that specific genes are functionally required for *in vivo *invasion and hematogenous dissemination in MDA-MB-231, as well as in patient-derived breast tumors. Finally, we used statistical analysis to show that the signature can significantly predict risk of breast cancer metastasis in large patient cohorts, independent of well-established prognostic parameters.

**Conclusions:**

Our data provide novel insights into, and reveal previously unknown mediators of, the metastatic steps of invasion and dissemination in human breast tumors *in vivo*. Because migration and invasion are the early steps of metastatic progression, the novel markers that we identified here might become valuable prognostic tools or therapeutic targets in breast cancer.

## Introduction

Breast cancer is one of the most frequent malignant neoplasms occurring in women in developed countries, and metastasis is the main cause of cancer-related death in these patients. The idea of personalized medicine and molecular profiling for prognostic tests has led to a plethora of studies in the past 10 years in search of genetic determinants of metastasis. Such studies have identified gene sets, or "signatures," the expression of which in primary tumors is associated with higher risk of metastasis and poor disease outcome for the patients. Early methods of analysis treated the tumor as a whole, so that the first molecular classification of tumors and identification of gene signatures associated with metastasis were all derived from whole pieces of tumor tissue [1-6]. These signatures were predictive of metastasis in patients and an important step toward applying these methods in clinical care. However, these signatures, mostly built to act as a general prognostic tool for the clinic, gave little information about the molecular biology of the different cell types comprising the tumor tissue and little insight into the specific mechanisms of metastasis. We now know that tumors are highly heterogeneous, that not all cells within a tumor are migratory and invasive, and that the tumor microenvironment gives spatial-temporal cues to tumor cells for invasion and metastasis [7]. In reality, only a small minority of tumor cells in the primary tumor is actually motile and capable of invasion and dissemination at any given time, as has been visualized in mouse and rat mammary tumor models with intravital multiphoton microscopy [8,9]. In addition, metastasis is a multistep process that involves the escape of cells from the primary tumor via either lymphatic or blood vessels, transport to and arrest in a target organ, or growth of metastases in the target organ [10]. Each of these steps is a multifactorial process, with potentially different tumor cell properties and molecules playing critical roles, and therefore each of these steps separately deserves detailed attention. More recent signatures give such emphasis in detailed analysis of the role of the microenvironment in metastasis [11], as well as analysis of the tissue tropism for metastatic growth [12]. The latter studies have been informative in prognosis of site-specific metastasis, as well as the cell biology behind the mechanisms of extravasation, homing, and colonization at the distant metastatic site [13-15]. However, little information is available about the crucial, potentially growth-independent, early steps of the metastatic cascade: migration, invasion, and entry of tumor cells into the systemic circulation.

We report for the first time a gene-expression profile for human breast tumor cells specific to the processes of invasion and migration in the primary tumor. We used orthotopic xenografts of MDA-MB-231 human breast tumor cells as our model, because this is an established breast adenocarcinoma cell line, widely used by the scientific community for studying *in vivo *metastasis based on its ability to grow orthotopic tumors, in mice, that spontaneously metastasize to other organs. Other established breast cancer cells lines metastasize in mice only in experimental settings (for example, via tail vein or intracardiac injection); however, these settings completely bypass the crucial and physiologically relevant steps of migration and invasion inside the primary tumor. Here, we show that specific genes from our signature are functionally required for *in vivo *invasion and hematogenous dissemination in mice bearing orthotopic tumors from human MDA-MB-231 cells, as well as orthotopic tumors in mice derived from patient primary breast tumors. We also show that this signature is predictive of distant metastasis in large patient cohorts, independent of other well-established clinical parameters. The present findings suggest novel mediators specifically for the early steps of metastasis, invasion, and hematogenous dissemination of breast tumors *in vivo*.

## Methods

### Cell culture

MDA-MB-231-GFP cells were cultured in DMEM (Invitrogen, Carlsbad, CA, USA) with 10% fetal bovine serum (FBS) (cell line generated by stable transfection of plasmid expressing GFP in parental ATCC line, as described in [16]).

### Animal models

All procedures were conducted in accordance with the National Institutes of Health regulations and approved by the Albert Einstein College of Medicine animal use committee.

For the MDA-MB-231 xenografts, a total of 2 × 10^6 ^MDA-MB-231-GFP cells per animal were resuspended in sterile PBS with 20% collagen I (BD Biosciences, Franklin Lakes, NJ, USA) and injected into the lower left mammary fat pad of SCID mice (NCI, Frederick, MD, USA). All experiments were performed on tumors that were 1 to 1.2 cm in diameter.

For the patient-derived xenografts: All human tumor tissue was received as discarded tissue (that is, excess tumor tissue after enough specimen was collected by the Weiler Hospital Anatomic Pathology Department for diagnostic tests). Because the tissue was not collected specifically for the proposed study and did not contain a code derived from individual personal information, no patient consent was required, as per institutional IRB approval. Tumor tissue was assigned a random number ID when received at the laboratory and implanted in mice within 2 to 3 hours of resection from the patient. The tissue was rinsed with sterile Hank's Balanced Salt Solution (HBSS; Invitrogen) cut in pieces of 2 to 3 mm and coated in matrigel (BD Biosciences, Franklin Lakes, NJ, USA). Two pieces of tumor were implanted surgically in both left and right lower mammary fat pads of SCID mice. The mice were supplemented with estrogen pellets (1.7 mg/pellet, 90-day release; Innovative Research of America, Sarasota, FL, USA), unless the tumor was already known to be ER-negative. The mice were monitored for growth for up to 9 months, at which time, if a tumor was not visible, they were euthanized. For the tumors that grew, *in vivo *invasion was measured, and then the tumor was used to passage to new mice (surgical procedure, same as before). Tumor cells were never passaged in culture or dissociated, but only propagated as tumor chunks *in vivo*. Part of each tumor and the lungs of the mice were fixed for histology analysis. Staining for human cytokeratins was performed with the CAM5.2 anti-cytokeratin antibody (BD Biosciences), as per the company's instructions. Staining was also performed in all tumors for ER, progesterone receptor (PR), and Her2 amplification. We found that the two ER^+ ^samples that successfully grew propagatable tumors in SCID mice lost their ER expression generally by the second passage (even when the mice were supplemented with estrogen). Other groups have successfully reported establishment of ER^+^-stable tumors in mice, but these either were derived from pleural effusions or used a different mouse strain [17,18]. At this time, we cannot be certain whether these technical differences would account for the establishment of stable ER^+ ^tumors, or whether this was a mere property of these two particular patient tumors that we tested.

For the blocking treatments, mice were injected intraperitoneally 4 hours before experiments with 100 mg/kg anti-IL8 antibody (MAB208; R&D Systems, Minneapolis, MN, USA), or 25 mg/kg of SB431542 (Tocris, Ellisville, MO, USA), NSC87877 (Tocris), NSC348884 (Sigma, St. Louis, MO, USA), or 10058-F4 (Sigma). Vehicle controls were the same quantities of DMSO (Sigma) for the SB431542, NSC348884, and 10058-F4 experiments, of isotype control IgG (BD Biosciences) for the anti-IL8 experiment, and of sterile water for the NSC87877 experiment. After each experiment, mice were euthanized, and the tumors were excised and fixed for further histologic analysis. Sections of all of the tumors from the treated mice were stained for H&E, as well as for Ki67 and cleaved caspase-3 as markers of proliferation and apoptosis, respectively. No significant differences were found between the vehicle control and inhibitor-treated mice for these markers, in the acute 4-hour treatments that were performed for these experiments to assay only for migration. For the MYC inhibition with small-molecule inhibitor 10058-F4 and to establish that the inhibitor indeed blocked proliferation *in vivo*, BrdU incorporation was also measured. Mice were injected intraperitoneally with 200 μl of BrdU (Sigma) of 10 mg/ml solution in sterile PBS 3 hours before killing, and then tumors were excised, fixed in formalin, and stained for anti-BrdU antibody with standard procedures. In brief, samples for immunohistochemistry (IHC) were sectioned at 5 µm, and deparaffinized in xylene followed by graded alcohols. Antigen retrieval was performed in 10 m*M *sodium citrate buffer at pH 6.0, heated to 96°C, for 20 minutes. Endogenous peroxidase activity was quenched by using 3% hydrogen peroxide in PBS for 10 minutes. Blocking was performed by incubating sections in 5% normal donkey serum with 2% BSA for 1 hour. Primary antibodies were rabbit polyclonal anti-Ki67 (VP-K451, Vector, 1:1,500), mouse monoclonal anti-BrdU (Roche, 1:400), and rabbit polyclonal anti-cleaved caspase 3 (Cell Signaling, 1:50). Tumor sections were stained by routine IHC methods, by using HRP rabbit polymer conjugate (Invitrogen), for 20 minutes to localize the antibody bound to antigen, with diaminobenzidine as the final chromogen. All immunostained sections were lightly counterstained with hematoxylin. For quantification, at least five random images were taken per tumor with at least three tumors per group, by using a Nikon Coolscope (at ×40 for Ki67 and BrdU stainings, and at ×20 for the cleaved caspase 3 stainings). Necrotic tumor areas were excluded from the analysis (no significant difference in overall necrosis was seen between treatments).

### *In vivo *invasion assay

Cell collection into needles placed into live anesthetized animals was carried out as described previously [19,20]. Migratory cells enter the needles only by active migration toward the chemotactic gradient. Cells are not passively collected in this assay, and the cells collected are not a biopsy sample, because a block is used to prevent passive collection of cells and tissue during insertion of the needle into the primary tumor. Cell migration and chemotaxis have been demonstrated to be required for cell collection [21]. After 4 hours of collection, the needles are removed, and the total number of cells collected is determined by DAPI staining. The chemoattractants used in this study include human recombinant EGF (Invitrogen) at final concentration of 25 n*M*, as well as 10% FBS serving as a general chemoattractant source. We controlled for the effects of technical aspects of our cell-collection method as described in Additional File 1.

### Intravasation assay

The number of circulating tumor cells was measured in mice bearing a tumor of 1 to 1.2 cm, as previously described [22]. In brief, blood was drawn from the right heart ventricle of anesthetized mice, and whole blood was plated in DMEM/20% FBS. Tumor cells were counted after 1 week. Cells counted from MDA-MB-231-GFP xenograft mice were GFP positive, confirming their identity as tumor cells. As a control, blood from non-tumor-bearing mice was plated as well, and absence of epithelial tumor cells was confirmed.

### Immunofluorescence

Migratory cells were isolated with the *in vivo *invasion assay, and after collection, they were extracted from the microneedles in a drop of ice-cold PBS on glass slides. Each needle content was carefully examined under a microscope to exclude needles from necrotic tumor areas, where cells could have entered the needle by passive flow and not by active chemotactic migration. The contents of successful needles were then transferred to a tube, spun down, and resuspended in 100 to 150 μl of 4% PFA (paraformaldehyde) in PBS to fix the cells immediately. Glass-bottom dishes (catalog number P35G-1.5-10-C; Mattek, Ashland, MA, USA) were coated with 0.05% PEI (polyethylenimine; catalog number P-3143; Sigma), and the fixed cells were added on the glass and allowed to stick for 20 to 30 minutes. The tumor from the same mouse was excised and mechanically dissociated on ice, and average primary tumor cells were isolated in the same way as they were isolated for the microarray samples and as described previously [16,23]. About 20K cells were also fixed immediately after preparation with 4% PFA and attached in PEI-coated glass-bottom (Mattek) dishes. After both cell populations were fixed and attached on dishes, standard immunofluorescence protocol was followed. In brief, cells were permeabilized by treatment with 0.1% Triton-X for 5 minutes, washed 3 times with PBS, incubated with blocking buffer PBS/1% BSA/1% FBS for 1 hour in RT, and then incubated with primary antibody to Smad2/3 (catalog number 610842; BD Transduction Laboratories, dilution 1:50) in PBS/1% BSA for 1 hour, washed 3 times with PBS/1% BSA, incubated with secondary antibodies and DAPI as a nuclear counterstain, and washed again 3 times with PBS/1% BSA. All samples were imaged by using a ×60 objective at an Inverted Olympus IX70 microscope equipped with a Sensicam QE cooled CCD camera. Processing and quantification of images was performed by using ImageJ software.

### RNA extraction, amplification, probe labeling, and microarray hybridization

RNA extraction, reverse transcription, SMART PCR amplification, microarray probe labeling, hybridization, and image collection were performed exactly as described in previous studies [23-25]. Four independent biologic repeats were used for the invasive tumor cells and the average primary tumor cells, respectively. Every sample was hybridized on one chip together with a common reference (human reference RNA from Clontech, amplified by using the same conditions as the experimental sample). Custom printed 27K Human cDNA microarray chips were used for the hybridization (NCBI GEO platform GPL15524).

### Quality control and significance analysis of microarrays

The scanned images were analyzed by using the software Genepix (Axon Instruments, Foster City, CA, USA), and an absolute intensity value was obtained for both the channels. Data filtering and global LOWESS normalization were done as described previously [24,25]. Statistical analysis was performed by significance analysis of microarrays (SAM) [26]. The data discussed in this publication have been deposited in the NCBI Gene Expression Omnibus [27,28] and are accessible through GEO Series accession number GSE37733. In total, 443 significantly differentially expressed transcripts were identified by SAM at a false discovery rate (FDR) of 10% when comparing migratory tumor cells with average primary tumor cells. Of these transcripts, 185 encode known protein products (gene list available in Additional File 2; italic font denotes genes with multiple spots).

### IPA and GSEA analysis of the human invasion signature

The Ingenuity Pathways Knowledge Base (IPA) version 8.7 was used to identify enriched functional gene networks and canonic pathways among differentially regulated transcripts of the human invasion signature [29]. The full 443-gene list that resulted from the SAM analysis of the microarrays was used for the IPA analysis. The *P *values were calculated by IPA by using a right-tailed Fisher Exact test. A cutoff of *P *< 0.05 was used for significance, as suggested by the software.

Gene-set enrichment analysis (GSEA) [30,31] was used to identify KEGG pathways upregulated in the human invasion signature. The full microarray dataset (after filtering and normalization) was used as input in the GSEA analysis. The KEGG pathways gene set was downloaded from the GSEA Molecular Signatures Database [32]. Statistical significance was assessed by using 1,000 gene-set permutations. A cutoff of FDR <25% was used for significance, as suggested by the GSEA team in the GSEA website.

### Knockdown by siRNA and transwell invasion assays

Small interfering RNAs for genes *SMAD2, IL8, PTPN11*, and *NPM1 *DONE! were purchased from Qiagen (validated FlexiTube siRNA, catalog numbers: SI02757496, SI02225902, SI02654960, and SO02654834). siRNA was resuspended to final 20 μ*M *concentration, according to manufacturer's instructions. siRNA was transfected into MDA-MB-231 cells by nucleofection (Lonza), according to the manufacturer's optimized protocol for the MDA-MB-231 cell line. Knockdown of each gene was confirmed with real-time PCR. As a negative control, a nontargeting sequence siRNA was used (Qiagen, catalog number 1027281), and we confirmed that this had no effect on expression of any of the genes tested in this study. Transwell *in vitro *invasion assays were performed by plating 25,000 MDA-MB-231 cells in the upper chambers of 8.0-μm pore size reduced growth factor Matrigel chambers or control noncoated chambers (BD Biosciences) in 0.5% FBS/DMEM. Cells were allowed to invade for 24 hours toward 10% FBS/DMEM, fixed with ice-cold methanol, and stained with 0.5% crystal violet. Two chambers per condition in at least three independent experiments were imaged at ×10, and four fields per chamber were counted and analyzed. Transwell assays for the siRNA-transfected cells were set up at day 3 after transfection, when knockdown was determined to be optimal. For the transwell assays with blocking treatments, the following concentrations of inhibitor or antibody were used in both the upper and bottom chambers (based on previous literature about each respective treatment): neutralizing anti-human IL8 antibody at 20 μg/ml, SB431542 at 10 μ*M*, NSC878887 at 50 μ*M*, and NSC348884 at 5 μ*M*. Each experiment was normalized to its appropriate control (equal amounts of nontargeting siRNA for all siRNA transfections, and equal amounts of DMSO, water, or control IgG for the inhibitors and neutralizing antibodies).

### Real-time PCR confirmation

Quantitative PCR analysis was performed as described previously [16], by using the Power SYBR Green PCR Core Reagents system (Applied Biosystems). For validation of microarray targets, the cDNA used as input for the PCR reactions was amplified with the same protocol as described earlier for microarray analysis (but from independent biologic repeats). Primer sequences are shown in Additional File 3. For validation of the siRNA experiments, RNA was extracted from at least three separate transfection experiments for each gene by using the Qiagen RNeasy Mini kit, and 1 μg of total RNA was reverse transcribed by using SuperScript II (Invitrogen) and oligo(dT) primers. Finally, 1 to 2 ng of single-stranded cDNA was used as input in the real-time PCR reactions. Each PCR reaction was performed in triplicate, and the mean threshold cycle (C_T_) values were used for analysis. GAPDH was used as a housekeeping gene control. Results were evaluated with the ABI Prism SDS 2.1 software.

### Biostatistics analysis of the human invasion signature

For the UNC232 cohort, patient gene-expression and clinical data published in [33] were downloaded from [34] (publicly available). For the NKI295 cohort, patient gene-expression and clinical data published in [3] were downloaded from [35] (publicly available). In both datasets, if multiple array probe sets referred to the same gene, the probe set with the greatest variation was selected to represent the gene. Clinical data associated with these cohorts are reported as recurrence-free survival for the UNC group and as metastasis-free survival for the NKI group. We used the top 80 regulated genes (by fold differential expression) in the human invasion signature for the analysis, trying to keep the gene lists as identical as possible for both UNC and NKI cohorts, considering that spots corresponding to some of our genes could not always be found on the original patient microarrays. Therefore, of these top 80 genes of the HIS, we were able to find the patient-expression data for 76 genes in the NKI295 database and the patient expression data for 79 in the UNC database (see "notes" column in spreadsheet of Additional File 2).

The method from Minn *et al*. [14] was used to investigate the relation between the human invasion signature and recurrence-free or metastasis-free survival in UNC232 and NKI295 cohorts. A training-testing method known as leave-one-out cross-validation was used to generate a risk index for each case. This risk index was defined as a linear combination of gene-expression values weighted by their estimated univariate Cox model regression coefficients. In each round, the gene-expression profile for each gene belonging to the invasion signature was used to fit the univariate Cox proportional hazards regression model in all cases minus one (training sample). The coefficients of these models were used to calculate the risk index later on the single test case that had been removed earlier. If a risk index was in the top 20^th ^percentile of the risk index scores of the training sample, then it was assigned to a high-risk group. Otherwise, it was assigned to a low-risk group. Repeating this procedure as many independent times as the number of patient cases, the risk-index value was determined for each case. All cases were assigned to a high- or low-risk group. Kaplan-Meier survival plots and log-rank tests were then used to assess whether the risk-index assignment was validated. To assess whether the association between our signature and metastasis-free survival was specific in the NKI295 cohort, we generated 1,000 random signatures of equal size to the HIS (that is, lists of randomly picked 76 genes) and tested their association with outcome by using the same method as detailed earlier. Multivariate Cox-proportional hazard regression modeling (SPSS) was used to determine the extent to which the HIS and other clinicopathologic parameters were independent prognostic indicators.

To estimate the similarity of the gene-expression pattern of the UNC232 cohort patients to the HIS, an *R *value was calculated for each subject in relation to the HIS by following the method of Creighton *et al*. [36]. The *R *value was defined as the Pearson's correlation between the HIS pattern (using "1" and "-1" for up- and downregulation, respectively) and the primary tumor's expression values, resulting in high *R *values for the tumors that tend to have both high expression of the upregulated genes and low expression of the downregulated genes in the human invasion signature. Before computing the *R*-value, the gene-expression values were centered on the centroid mean of the comparison groups of interest. The *R *value for each patient was then calculated, plotted, and grouped by breast cancer subtype.

### Statistical analysis of mouse experimental methods

All statistical analyses, unless otherwise stated, were assessed by using unpaired, two-tailed Student *t *test, assuming equal variances. Differences were considered significant if the *P *value was <0.05. For the intravasation assay, the Mann-Whitney Wilcoxon rank sum test was used in addition to the Student *t *test.

## Results

### Gene-expression profile of migratory human tumor cells *in vivo*: the human invasion signature

We previously showed that we can collect the migratory cells from MDA-MB-231 primary tumors in response to epidermal growth factor (EGF) or colony-stimulating factor-1 (CSF1) by using an *in vivo *invasion assay [16]. In brief, microneedles containing a chemoattractant are placed in primary tumors while the tumor-bearing mouse is alive and under anesthesia. This creates a chemotactic gradient similar to physiological stimuli inside the primary tumor, shown to initiate tumor cell invasion. Indeed, we previously reported that chemotaxis and active migration are required for the tumor cells to enter the microneedles [21]. Thus, this assay tests the cells' ability *in vivo *to undergo chemotaxis toward a chemokine gradient, to invade through the tumor matrix, and finally to migrate over long distances toward the source of the gradient [21]. For brevity, the tumor cells collected with this assay will be hereafter called "migratory tumor cells." With this assay, we recently showed that the invasive properties of the MDA-MB-231 human breast adenocarcinoma cells differ *in vitro *and *in vivo*, because of a TGF-β-initiated autocrine CSF1/CSF1R loop that occurs in the tumor microenvironment [16]. We also showed that this hypermotile tumor-cell subpopulation spontaneously expresses an invasion-specific isoform of Mena (MenaINV), which is the hallmark of migratory tumor cells in mammary tumors [7,37,38]. This emphasizes the importance of isolating the migratory tumor cells directly from the primary tumor *in vivo*, to understand their full potential and characteristics.

**Figure 1:**
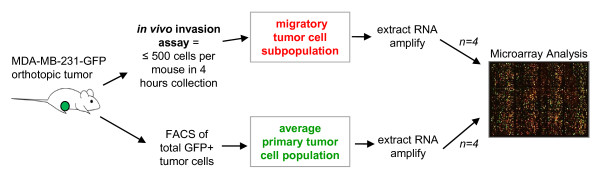
**Study design for the derivation of the human invasion signature (HIS)**.Schematic of experimental procedures for the selective profiling of migratory tumor cells *in vivo*. Migratory/invasive tumor cells were isolated from live primary tumors based on their chemotactic and highly motile properties toward known chemoattractants. The whole or "average" primary tumor cells were isolated with fluorescence-activated cell sorting (FACS) based on the tumor cells stably expressing green fluorescent protein (GFP). Both cell populations analyzed were >95% pure tumor cells (detailed discussion in Additional File 1). Comparative gene-expression analysis with microarrays was then used to derive a signature specific to *in vivo *migration and invasion of breast tumor cells. Methods and technical controls are discussed in more detail in Methods and in Additional File 1.

With this *in vivo *invasion assay, we isolated the migratory tumor cells from orthotopic MDA-MB-231 tumors and then compared their gene-expression profile by microarray analysis with the total or "average" primary tumor cell population, which is primarily nonmigratory (Figure 1; technical controls discussed in more detail in Additional File 1). Overall, 443 transcripts were found to be significantly altered in the migratory tumor cells, of which 185 were annotated genes with known protein products (gene list in Additional File 2). We define this gene list as the human invasion signature (HIS).

To gain insight into the biologic properties of the migratory breast tumor cells, Ingenuity Pathway Analysis (IPA) was used first to rank enriched functional categories of gene networks relating to the transcripts regulated in the HIS. Table 1 shows the top five most significantly upregulated and downregulated functions related to the gene networks of the HIS, along with the list of the corresponding genes in each function network. The most highly upregulated gene networks in the migratory tumor cells are involved in regulating the functions of DNA replication and repair, embryonic and tissue development, and cellular movement. Interestingly, an independent study of tumor-associated macrophages (TAMs) recently showed that invasive macrophages isolated from primary mammary tumors of transgenic mice also demonstrate a resemblance in their genetic profile to embryonic macrophages when compared with the general TAM population [39]. These data suggest that a recapitulation of developmental programs may be adopted by the breast tumor cells and their partner macrophages during invasion and migration in primary tumors. In the functions that are downregulated in the migratory tumor cells, cell cycle and cell death were among the most significant (Table 1). This result is consistent with previous results that showed that migratory cells isolated from a transgenic mouse mammary tumor showed decreased proliferation and apoptosis compared with the average primary tumor cells, resulting to an increased resistance to chemotherapy [40].

**Table 1:** Significant upregulated and downregulated functional gene networks of the migratory breast tumor cells

Rank	Score	Function network	Genes regulated in function network
**Upregulated**			

1	48	DNA replication and repair	*ALDOA, CDC25A, CDK1, CKS1B, CSDE1, DAZAP2, DBP, EMP1, FOXM1, IFI16, NCL, NONO, NPM1, PMAIP1, POLR2G, PTAFR, S100A11, SET, SF3B2, SKP1, SLC20A1, TRIM32, UBC, XRCC5*

2	36	Embryonic and tissue development	*ACVR1B, ARHGDIB, CAP1, CAV1, CDC42, DDX24, FADD, GLO1, IL8, KLF11, LSM3, MSN, NCAPD3, PPM1A, PTPN11, RPS6, SMAD2, SNRPD1, SNRPD3, SNTB2, UTRN, VAMP7, YWHAE*

3	33	Cellular movement and development	*ARHGAP11A, CNN3, ITGAE, MRPL27, OSGEP, PHACTR2, PRDX5, RFC3, RPL30, RPL37, RPL12, SNRPD3, TUBA1A, TUBA4A, TXNDC9, UBE2D3, ZNF184*

4	33	Cell-to-cell signaling and interaction	*ACAP2, ASPH, CALU, COX7B, GARS, IMPDH2, ISLR, NOP10, PRDX3, RABIF, RPL11, RPL19, SDHD, STRBP, USP13, WBP5, ZNF207*

5	27	Cellular assembly and organization	*ATP5G1, ATP5I, ATP6V0A1, DDAH1, DGUOK, ERH, FMOD, MYL12A, PSMB2, PSME2, SF3B14, STXBP2, TBCA, UQCR10, VAMP7*

**Downregulated**			

1	44	Nervous system development and function	*AKAP13, BBS2, CEACAM6, CHP, CREB1, DLG1, HSPB6, IL11, IL32, INA, ITGB3BP, NUP62, PNRC1, S1PR2, SH3BP2, SLC2A3, SLCO1B3, STAR, TNFRSF9, TRIM13, VDR*

2	31	Cell death and cell cycle	*ACRBP, ATP8A1, BCL7B, DOC2B, GOSR1, IREB2, MIB2, NDUFB2, PSMD5, RASA4, RPL37, SLC2A3, TGFB1I1, TNF, TP53I3, TP53INP1, TST, TTF1, YTHDC1*

3	22	Hematologic disease	*CHP, CNN2, F11, FRG1, GATAD2B, HSDL2, KCNJ9, POFUT1, SGCB, TSPAN14, ZFC3H1, ZNF165*

4	19	Protein synthesis and cell morphology	*EIF4A1, EPB49, HEBP2, MACF1, MLL4, MPRIP, MYO1C, RAG1AP1, TES, UBR5, ZNF790*

5	18	Drug and nucleic acid metabolism	*IL10RB, MDM2, NAIP, PIP5K1C, PPFIBP1, SLC25A37, SLC2A3, SLC38A2, SNRNP70, STK25, ZNF331*

The human invasion signature (HIS) was analyzed for significant regulated functions by using Ingenuity Pathway Analysis. The genes associated with each function network shown in the last column are significantly regulated in the HIS. Score: negative exponent of p value calculated by a right-tailed Fisher Exact test (calculates the likelihood that the Network Eligible Molecules that are part of a network are found therein by random chance alone).

### Validation of specific genes from the human invasion signature

**Figure 2:**
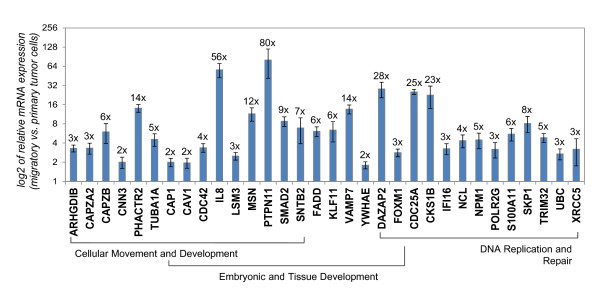
**Validation of specific genes upregulated in the migratory breast tumor cells**. mRNA expression of genes from the top three significant upregulated function networks in Table 1 was assessed with real-time polymerase chain reaction (PCR) in independent biological repeats of migratory tumor cells versus average primary tumor cells from MDA-MB-231 breast tumors. Genes are grouped by function, as determined by Ingenuity Pathways Knowledge Base (IPA) and Gene Ontology annotations. Bars, relative average mRNA expression of migratory tumor cells compared with average primary tumor, log_2_-transformed scale for ease of display. The linear fold-upregulation for every gene is shown at the end of every bar. Error bars: SEM, *n *= 6, *P *< 0.05 for all data shown in this graph (Student *t *test).

We went on to validate the gene-expression changes found in the HIS by real-time RT-PCR in independent biologic repeats of migratory tumor cells and average primary tumor cells isolated from MDA-MB-231 tumors. We specifically concentrated on the genes from the three most significantly upregulated functional networks identified by IPA (Table 1). It is our hypothesis that these genes will be most likely to have central roles in invasion and metastasis of the breast tumor cells, and therefore most likely to be more useful and relevant as potential prognostic markers and/or therapeutic targets. We confirmed the upregulation of the majority of these genes with independent biologic repeats, and in most cases, the fold change of the mRNA expression was actually underrepresented in the DNA microarrays (Figure 2). We subgrouped the genes by function, according to the IPA results, as well as Gene Ontology annotations. The biggest overlap for genes having double annotated functions was seen between the "embryonic and tissue development" and the "cellular movement" gene networks (Figure 2), with more than half of the genes shared between the two functions. Some of the upregulated genes confirmed here have well-established roles in invasion and metastasis, such as *SMAD2 *[41], *CDC42 *[42], and *VAMP7 *[43]. Other genes have been correlated with tumorigenesis, such as *CDC25A *[44], *PTPN11 *[45], and *IL8 *[46], but have not been extensively studied in regard to migration and invasion of breast tumor cells. A potential link between DNA replication and repair genes and *in vivo *invasion is also evident, with genes such as nucleolin (*NCL*) and nucleophosmin (*NPM1*) greatly upregulated in the migratory breast tumor cells. Of additional interest, for some of the genes confirmed here, such as *DAZAP2 *and *KLF11*, very little is known about their involvement in cancer and metastasis. However, DAZAP2 is essential for neural patterning in *Xenopus laevis *embryos [47], and KLF11 is an activator of embryonic and fetal beta-like globin genes [48], again pointing to a connection between regulation of embryonic development and cancer invasion. Overall, the HIS has identified novel genes that could potentially have important roles in the regulation of invasion and migration of breast tumor cells *in vivo*.

We further analyzed these top upregulated genes by using the IPA software to create a regulatory network map. Because the DNA replication and repair network showed minimal overlap with the other networks, a separate map was drawn (Additional File 4). For the embryonic-development and cell-movement networks, a common map was drawn, because most of their genes were shared. Interestingly, one of the central nodes of interaction for the top upregulated genes in the HIS was TGF-β (Additional File 5), a pathway that was also found statistically enriched in the HIS by both IPA and Gene Set Enrichment Analysis (GSEA) toward curated canonic pathway gene sets (Additional File 6). We recently showed that TGF-β is the microenvironmental factor that initiates an autocrine invasion phenotype for human breast tumor cells by upregulating the expression of the colony-stimulating factor-1 receptor (CSF1R) in the MDA-MB-231 breast tumor cells *in vivo *[16]. This is consistent with our current results, in which TGF-β is not regulated itself in the migratory tumor cells, but it is a central signal for their invasive gene profile. Finally, an enriched TGF-β signaling profile is also consistent with the hypothesis that the tumor cells recapitulate developmental gene-expression programs while in the process of migration, as TGF-β is known to play roles in several stages of mammary gland development [49,50].

### Inhibition of specific targets from the human invasion signature abrogates invasion and hematogenous dissemination *in vivo *(2)

**Figure 3:**
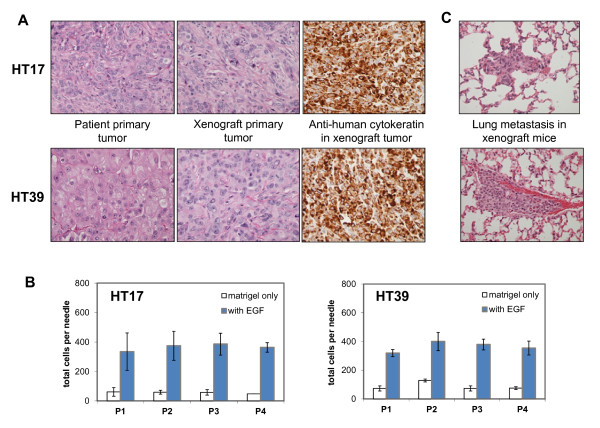
**Histologic and metastatic properties of the patient-derived orthotopic breast tumor xenografts**. **(A) **For the HT17 and HT39 patient tumors, representative images are shown here of (from left to right) primary tumor from the patient of origin (H&E), primary tumor in the xenograft (H&E), and staining of the xenograft tumor with a human-specific anti-cytokeratin antibody (immunohistochemistry, brown). Magnification ×40. **(B) ***In vivo *invasion assay for HT17 and HT39 xenograft tumors to an EGF gradient, passages 1 through 4 (P1-P4). Invasion to a gradient of serum (FBS) showed similar results. The number of migratory cells remains similar over passages (*P *= 0.47 for HT17, *P *= 0.82 for HT39, by one-way ANOVA). Results are plotted as average number of cells per microneedle. Error bars: SEM, *n *≥ 5 mice. **(C) **Representative images of spontaneous lung metastasis of the xenograft orthotopic tumors (H&E). Magnification ×40.

To complement the results from MDA-MB-231-derived tumors and to validate a potential clinical significance for our results, we developed xenografts from patient-derived breast tumor tissue collected from surgical resections and surgically implanted in the mammary fat pad of SCID mice. We implanted in total more than 30 patient breast tumor tissue samples in mice, with a growth take rate of approximately 28% (Table 2). Other studies of patient breast tumor implantation have reported somewhat higher take rates. However, these either were not orthotopic and used the abdominal fat pad or subcutaneous implantation sites, or included samples from pleural effusions, which overall have a higher take rate in mice [17,51,52]. We used only primary tumor tissue (not pleural effusions or tissue from metastatic sites), and we implanted specifically in the mammary fat pad, to have a more relevant microenvironment for breast tumor growth and a clinically relevant route for invasion and dissemination from the primary tumor site. As our study focused on invasion in the primary site of metastatic breast cancer, our goal was to find those patient samples that would establish patient-derived tumors that are stably propagatable in mice, have a tumor latency of less than 6 months, and are invasive and metastatic as a xenograft tumor. We chose to focus on tumors HT17 and HT39, which among our samples were the most stable, invasive, and metastatic (Additional File 7). We confirmed that even after up to four passages in mice, tumors HT17 and HT39 exhibited histology similar to the patient they were derived from, remained human in origin, as well as retained their invasive and metastatic potential (Figure 3).

**Table 2:** Development of patient-derived breast tumor xenografts in SCID mice

	Total	ER^+^	ER^-^	Triple negative
Patients samples received	29	17	12	7

Samples that grew tumors in mice after first implantation	8	4	4	4

Take rate	27.59%	23.53%	33.33%	57.14%

Samples that established a stable and propagatable tumor in mice (successful growth in subsequent passages)	6	2	4	4

Stable take rate	20.69%	11.76%	33.33%	57.14%

Numbers of patient samples implanted in the mammary fat pad of SCID mice and take rates for successful growth in the mice. For some of the samples, a tumor grew only on the first implantation. We call stable take-rate the percentage of samples that established tumors in mice that were capable of growing tumors in subsequent passages.

**Figure 4:**
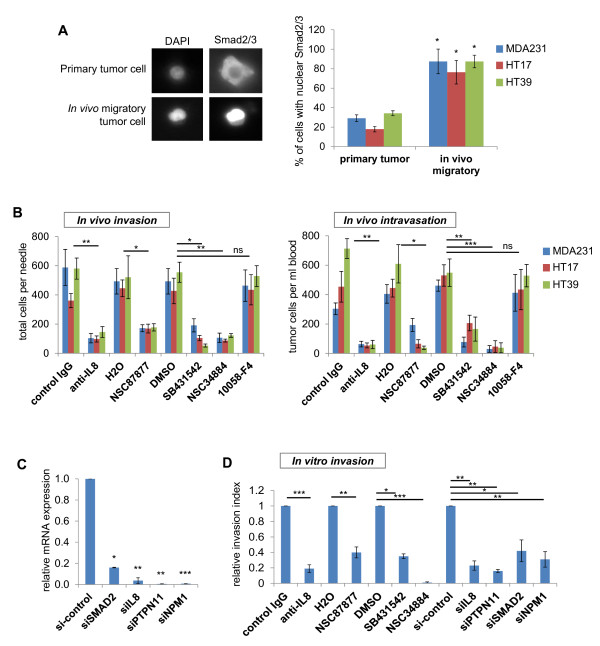
**Functional validation of specific targets from the HIS in human breast tumors *in vivo***. **(A) **Migratory and average primary tumor cells were isolated *in vivo *from MDA-MB-231 as well as the patient-derived HT17 and HT39 tumors. Cells were fixed immediately after collection and immunostained for total Smad2/3 complex, with DAPI used as a nuclear counterstain. A representative image for a cell with cytoplasmic Smad2/3 staining from the primary tumor samples and a cell with nuclear accumulation of Smad2/3 from the migratory cell samples is shown. Quantification of total results is shown in the graph, for which the average percentage of cells with nuclear Smad2/3 accumulation over total number of cells (by DAPI count) was calculated for each xenograft. Error bars: SEM, *P < 0.05 (Student *t *test), *n *= 10 to 50 cells per sample; samples from at least three different mice. *(***B) ***In vivo *invasion and intravasation were measured in mice bearing either orthotopic MDA-MB-231-GFP tumors (MDA231) or patient-derived HT17 and HT19 tumors, shortly after treatment with specific inhibitors or blocking antibodies. *In vivo *invasion is plotted as average number of migratory cells collected per microneedle. Intravasation is plotted as average number of circulating tumor cells per milliliter of blood. Results are shown for mice that received treatment with either vehicle control or specific inhibitor: neutralizing antibody specific to human IL8, PTPN11 specific inhibitor NSC87877, TGF-β receptor-specific inhibitor SB431542, NPM1-specific inhibitor NSC34884, or MYC-specific inhibitor 10058-F4 (negative control). Bars, average number of cells; error bars: SEM, *P < 0.05; **P < 0.01; ***P < 0.001; ns, not significant (Student *t *test for each condition relative to its vehicle control); *n *≥ 6 microneedles from at least four mice for the *in vivo *invasion assay; *n *≥ 6 mice for the intravasation assay. **(C) **mRNA expression of MDA-MB-231 cells transfected with siRNA for genes *SMAD2, IL8, PTPN11, NPM1*. Shown is expression for each target gene by its respective siRNA relative to the nontargeting control siRNA (si-control). Error bars: SEM, *P < 0.05; **P < 0.01; ***P < 0.001 (Student *t *test); *n *= 3 separate experiments for each siRNA. **(D) ***In vitro *invasion over Matrigel-coated transwells was measured for MDA-MB-231 cells, either transfected with siRNA to the genes indicated or with specific inhibitors or blocking antibodies. Shown is the relative invasion for each condition toward the appropriate control. Error bars: SEM, *P < 0.05; **P < 0.01; ***P < 0.001 (Student *t *test); *n *= three separate experiments for each condition with duplicate transwells per experiment.

Unsupervised analysis of the HIS gene-expression profile pointed to TGF-β as a central regulatory node of the top upregulated genes of our signature, although TGF-β was not itself upregulated in the *in vivo *migratory tumor cells (Additional Files 5 and 6). We sought to test directly at the protein level whether indeed TGF-β signaling was enriched in the migratory tumor cells *in vivo *compared with the primary tumor overall. For this, we isolated migratory tumor cells from MDA-MB-231 tumors, as well as the patient-derived primary breast tumors HT17 and HT39 described earlier. For comparison, the average primary tumor cell population was isolated from the same mice. Cells from both populations were fixed in suspension immediately after collection, to preserve their signaling status at that moment without adjustment due to plating and adhering to tissue-culture dishes. Fixed cells were immunostained with specific antibodies to Smad2/3 complex, which accumulates in the nucleus when the TGF-β pathway is active. We found that 80% to 100% of the migratory tumor cells showed nuclear accumulation of Smad2/3 compared with only about 20% to 30% of the average primary tumor in all three breast tumors tested (Figure 4A). These data indicate that TGF-β signaling is active in tumor cells while they are in the process of migrating and invading *in vivo *in human primary breast tumors.

We next sought to test the requirement of specific genes from the HIS in the early steps of metastasis, invasion, and dissemination *in vivo*. More effectively to model a potential clinical approach, and to avoid experimental artifacts in tumor growth resulting from shRNA viral infections of the primary breast tumor cells, we evaluated the effect of brief injection of specific pharmacologic inhibitors or neutralizing antibodies into mice with established tumors. We focused on TGF-β as a central regulator of the *in vivo *migration phenotype, as well as selected highly upregulated genes from the top three functional gene networks (Figure 2). We selected our targets with three general criteria: genes that were highly upregulated by the real-time PCR validation of Figure 2, that would represent the top three upregulated functional networks of Table 1 and for which specific inhibitors were commercially available. Specifically we targeted *IL8*, *PTPN11, and NPM1*, because they were highly upregulated, and because they appear as functional central nodes of their respective gene networks (Additional Files 4 and 5). IL8 (or CXCL8) was originally cloned as a factor attracting and activating neutrophils, eosinophils, and T lymphocytes [46], and as such, it has been shown to enhance tumor angiogenesis and growth through recruitment of neutrophils to the primary tumor site [53]. IL8 stimulation has been shown to promote invasion of breast tumor cell lines *in vitro *through reconstituted matrices [53,54], but its role in tumor cell migration and invasion *in vivo *has not been tested. *PTPN11 *(which encodes for the phosphatase Shp2) was first found as a gene of which germline mutations are linked to the developmental disorder syndromes Noonan and LEOPARD [55]. Somatic mutations in this gene are also associated with several types of human malignancies, most notably, juvenile myelomonocytic leukemia. In relation to the mammary gland, a conditional deletion of *PTPN11 *in transgenic mice showed impaired mammary gland development and morphogenesis of the alveolar structures [56]. PTPN11 upregulation has been noted in infiltrating ductal carcinomas [57], its activity has been implicated in integrin signaling during *in vitro *migration through Matrigel [58], and a recent report suggests a function for PTPN11 in tumor-initiating cells maintenance [59]. As far as *NPM1 *is concerned, mutations in this gene drive tumorigenesis in acute myeloid leukemia (AML) [60-62], but its role in solid tumors has been controversial [63-65]. Phosphorylated NPM1 is recruited to sites of DNA damage, whereas a nonphosphorylable mutant causes failure of DNA repair [66]. Again, its role in breast cancer invasion and dissemination has not been tested to date.

We used for our experiments small-molecule inhibitors that showed specificity for these targets, as evident from the literature: SB431542 (a small-molecule inhibitor shown to be specific for the TGF-β receptor TGFBR1 *in vitro *and *in vivo *[67,68]), NSC87877 (a small-molecule-specific inhibitor shown to be selective for PTPN11 at five- to 400-fold over other protein tyrosine phosphatases, such as PTP1B and LAR [69]), NSC348884 (a small-molecule inhibitor of NPM1 oligomerization and thus its active state [70]), as well as a neutralizing monoclonal antibody specific to human IL8 (tested with ELISA for cross-reactivity with other cytokines). Because the focus of our study is migration and invasion, a brief drug treatment of only 4 hours was given to the mice before experimental assays so that only the specific effect on migration and invasion can be measured without any long-term effects on tumor growth. We measured invasion by count of total cells that show chemotaxis and invade in the primary tumor toward a gradient source (EGF or FBS as a general chemoattractant source) with the *in vivo *invasion assay. We measured intravasation and hematogenous dissemination by count of circulating tumor cells (CTCs) in the total blood of tumor-bearing mice. When the inhibitors or neutralizing antibodies were injected into the tumor-bearing mice, *in vivo *invasion and intravasation (that is, the number of CTCs) were significantly inhibited compared with each respective vehicle control, in both MDA-MB-231 tumors and the patient-derived HT17 and HT39 tumors (Figure 4B). No significant difference in overall cell death was observed by histology in the treated tumors with the 4-hour brief treatments, suggesting that the inhibition seen is specific to migration. To mitigate potential concerns regarding specificity of the small-molecule inhibitors, we also directly targeted these pathways with siRNAs *in vitro *to confirm that their inhibition affected migration. Overall, siRNA to the genes *SMAD2 *(as a downstream effector of TGF-β signaling, also upregulated in the HIS, as shown in Figure 2), *IL8*, *PTPN11*, and *NPM1 *were significantly effective in knocking down expression of their respective target genes compared with a nontargeting siRNA control (knockdown by 84%, 96%, 99%, and 99%, respectively) (Figure 4C). In MDA-MB-231 cells, *in vitro *invasion through Matrigel-coated chambers was significantly inhibited by both the inhibitors/ blocking antibodies used earlier and by the siRNAs to each gene (Figure 4D), suggesting that the inhibitory effect observed is specific to the genes targeted. These data indicate that the genes identified by the HIS are potentially important mediators of breast cancer invasion and dissemination.

As a negative control, we used an inhibitor to a target that was not identified by the HIS. We chose to inhibit *MYC*, a known oncogene recently identified as a master regulator of expression of "poor-outcome" cancer signatures [71]. As hypothesized, brief treatment with 10058-F4, a small-molecule inhibitor of Myc-Max interaction [72], did not significantly alter either *in vivo *invasion or hematogenous dissemination in the human breast tumors (Figure 4B). BrdU incorporation (a proliferation marker) was significantly reduced in these same tumors, indicating that the inhibitor was indeed functional *in vivo *(see Additional File 8). Most of the published signatures to date are isolated from bulk tumor samples, and therefore represent "whole-picture" information about the metastatic process, a summary of invasion, dissemination, growth/proliferation, and stromal patterns of expression. *MYC *is a central oncogene that is required for carcinogenesis, as well as growth of metastatic lesions after the disseminated tumor cells have reached the target organ, and therefore, it is not surprising that it is a central regulator of earlier published signatures. Our results, however, show that *MYC *is not required for the isolated process of invasion, further suggesting that the HIS is a gene signature specific to the early metastatic steps of migration and invasion inside the primary tumor.

### The human invasion signature has prognostic value in breast cancer patients

**Figure 5:**
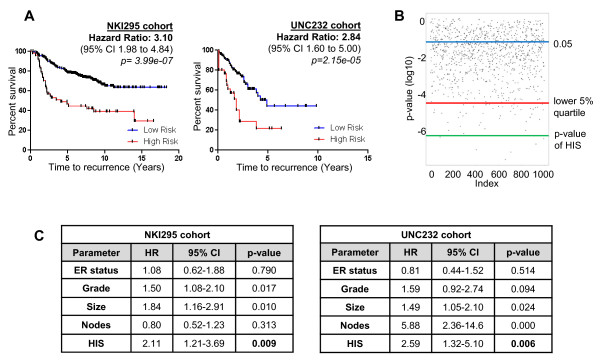
**The human invasion signature (HIS) is prognostic of clinical outcome in breast cancer patient cohorts**. **(A) **Metastasis-free survival Kaplan-Meier analysis on cases identified as high and low risk by the HIS in the NKI295 cohort. Hazard ratio, 3.10; 95% CI, 1.98 to 4.84; *P *= 3.99e-07 (log-rank test). Also shown is the recurrence-free survival Kaplan-Meier analysis on cases identified as high and low risk by the HIS in the UNC232 cohort. Hazard ratio, 2.84; 95% CI, 1.60 to 5.00; *P *= 2.15e-05 (log-rank test). **(B) **One thousand signatures of equal size to the HIS were generated by picking random genes from the genome, and their association to distant metastasis in the NKI295 cohort was calculated. In the scatterplot shown here, each dot represents the *P *value calculated for each of the random signatures. Blue line, *P *value of 0.05; red line, *P *value cutoff for the best 5% random signatures (*P *= 2.41e-05); green line, *P *value for the HIS (*P *= 3.99e-07). **(C) **Multivariate Cox-Proportional Hazard Regression Analysis of the HIS in the NKI295 and UNC232 cohorts, incorporating established clinical parameters. HR, hazard ratio; CI, confidence interval.

We next sought to determine whether the HIS has prognostic value in determining metastatic risk for patients with breast cancer. We investigated the association between metastasis-free or recurrence-free survival and the gene-expression profiles of the HIS for breast cancer patients from publicly available databases. We used two databases for our analysis, one from a NKI cohort study (NKI295) [3] and one from a UNC cohort study (UNC232) [33]. For this statistical analysis, we used a subset of the HIS that contained the top most differentially expressed 75 to 80 genes by fold-expression (gene list in Additional File 2). This list also contains the genes validated in Figure 2 and 2predicted to have roles in the top significant upregulated networks (Table 1). Our rationale was that, because these datasets are derived from whole pieces of tissue and therefore have a significant gene-expression contribution from both stromal and nonmotile tumor cells, the highest gene-expression changes are more likely to be observed above the noise and across multiple patients. Expression of this subset of genes of the HIS significantly separated breast cancer patients with increased risk of distant metastasis in the NKI295 cohort and increased risk of overall recurrence in the UNC232 cohort (Figure 5A), with hazard ratios of 3.10 (95% confidence intervals, 1.98 to 4.84; *P *= 3.99e-07) and 2.84 (95% CIs, 1.60 to 5.00; *P *= 2.15e-05), respectively. It was recently reported that most random signatures >100 genes can significantly predict outcome in the NKI295 cohort, with a significance of *P *< 0.05 [73]. Therefore, as a control, we compared the HIS with 1,000 random signatures of identical size and confirmed that the HIS has a much more specific association to patient outcome in this cohort than the best 5% random signatures (Figure 5B).

To determine whether the HIS carries additional prognostic information beyond variables commonly used in the clinical practice, or whether it is merely a surrogate readout for previously established risk factors, we performed a multivariate Cox proportional hazard regression modeling. When we incorporated tumor grade, lymph-node status, tumor size, and ER status, the HIS remained a significant independent predictor of outcome in both the NKI295 and the UNC232 cohorts (*P *= 0.009 and *P *= 0.006, respectively; Figure 5C).

**Figure 6:**
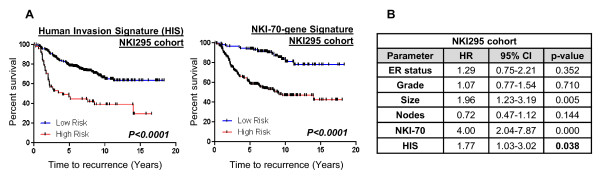
**Comparative analysis of the human invasion signature (HIS) with the NKI-70-signature**. **(A) **Metastasis-free survival Kaplan-Meier analysis on cases identified as high and low risk by the HIS in the NKI295 cohort (*P *< 0.0001). The graph is repeated here from Figure 5A for ease of comparison. Also shown is the metastasis-free survival Kaplan-Meier analysis on cases identified as high and low risk by the NKI-70 gene signature in the NKI295 cohort (*P *< 0.0001). **(B) **Multivariate Cox proportional hazard regression analysis was performed to evaluate the relation between the HIS and distant metastasis in the NKI295 cohort, incorporating relevant clinical variables as well as the NKI-70 signature (HR, hazard ratio; CI, confidence interval). The NKI-70 signature is a strong predictor, which is expected, because this signature was derived from this same cohort. However, the HIS is significant even in the presence of the NKI-70 signature, indicating that it contains additional prognostic information for this cohort beyond that captured by the NKI-70 signature.

Because many reported prognostic signatures can identify substantially overlapping groups of patients, we wanted to determine whether the HIS was an independent predictor of poor outcome when a well-established signature was included in the model. The NKI-70-gene signature is one of the earliest published signatures in the literature [4] and has resulted in the first FDA-approved microarray-based prognostic test for metastasis risk prediction in breast cancer (Mammaprint) [74]. We compared the HIS with the NKI-70-gene signature in the NKI295 cohort and found that both signatures performed comparably in selecting a group of patients with significantly poorer outcomes (Figure 6A). A difference between the two signatures is that the initial slope of the high-risk patients identified by the HIS is significantly steeper (Figure 6A) (*P *= 0.0258, by the Grehan-Breslow-Wilcoxon test), suggesting that the HIS may identify patients at higher risk of early metastasis. We then performed an additional multivariate Cox proportional hazard regression analysis incorporating the NKI-70-gene signature (Figure 6B). The NKI-70-gene signature was a strong predictor of metastasis in the NKI295 database, a result expected because it was derived from this same cohort. However, even in the presence of the NKI-70-signature, the HIS remained an independent predictor of distant metastasis (*P *= 0.038), suggesting that our signature carries significant prognostic information beyond that captured by the NKI-70-gene signature.

**Figure 7:**
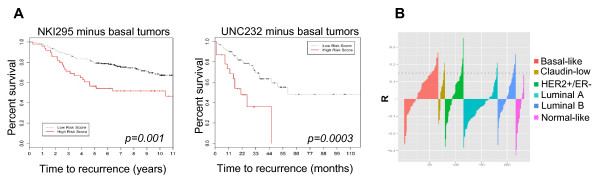
**The prognostic significance of the human invasion signature (HIS) is not confined to basal-like breast tumors**. **(A) **The HIS remains prognostic of outcome in patient cohorts after exclusion of basal-like tumor patients. Cox proportional hazards model analysis was repeated for the NKI295 and the UNC232 cohorts, excluding the patients with the basal-like breast cancer subtype. *P *= 0.00147 for NKI and *P *= 0.000345 for UNC (log-rank test). **(B) **A Pearson correlation *R *value was calculated to assess the relation between the HIS gene-expression pattern and the gene expression of each tumor in the UNC232 database. In the plot shown, *R *values for all patients are clustered by breast cancer subtype. *R *values above the dotted line are significant at *P *< 0.05. Patients with a gene-expression pattern positively correlated to the HIS appear in multiple breast cancer subtypes.

Because the microarray analysis was based on MDA-MB-231 tumors, a triple-negative basal-like breast cancer cell line [75], a concern was that the signature might be prognostic because it simply identifies the basal tumors, which are known to have a worse outcome [76]. To investigate this, we repeated the Cox proportional hazards model analysis, completely excluding the basal tumors from both cohorts, and again found that the HIS was prognostic of recurrence and metastasis in the patients of the remaining subtypes (Figure 7A). We also performed a correlation analysis of the HIS gene pattern to the gene expression of individual patients in the UNC232 cohort (method as performed previously for this cohort in reference [36]), and found that our signature does not identify with the gene pattern of any single breast cancer subtype (Figure 7B). Our data suggest that the migratory cells that we analyzed in this study are the tumor cells that will most likely invade and disseminate to form distant metastasis in patients. Therefore, patients with enriched numbers of these cells in their primary tumors are at higher risk for developing early metastasis or recurrence, regardless of tumor subtype.

## Discussion

In this study, we derived a unique invasion gene signature that we expect will reveal important information about novel mediators of the early steps of breast cancer metastasis: migration and invasion in the primary tumor. Our results show that the migratory human breast tumor cells, in their mRNA expression, share similarities with cells undergoing embryonic and tissue developmental programs, and that TGF-β signaling is a central regulator for this phenotype. An unexpected finding in our study was the upregulation of DNA replication and repair genes in the migratory breast tumor cells. Whether this is a parallel feature or an active contributor to the migratory abilities of the tumor cells is currently unknown and the subject of further future investigation in our laboratory. In the present study, we showed, by using small-molecule inhibitors, that the TGF-β pathway, as well as three of the top upregulated genes from our gene-expression profile, are functionally required for invasion and tumor cell dissemination *in vivo *in both cell-line and patient-derived primary breast tumors. Finally, we showed that expression of the human invasion signature is significantly associated with metastasis-free survival in breast cancer patients and predicts poor outcomes independent of other well-established prognostic factors. Of course, for technical reasons, the patient-derived tumors we used for our functional validation studies were triple-negative, and therefore we cannot exclude the possibility that our results may be more relevant for metastasis of triple-negative breast cancer. However, our statistical analysis of public patient cohorts shows that the HIS is a significant predictor of metastasis-free survival in other breast cancer subtypes. When taken together, these data imply that, although the HIS was derived from MDA-MB-231 tumors, our main observations have the potential to be broadly applicable to multiple types of human breast cancers.

In the past, an invasion signature was identified in MTLn3 rat mammary tumor xenografts and MMTV-PyMT transgenic mammary tumor mice [24,25]; however, the human invasion signature consists of a unique gene list that is not evident in the rat and mouse tumor models. For example, IL8, one of the highest upregulated genes in our signature, does not have a clear homologue in mice and rats and therefore was not previously discovered by using the rodent models. A strong correlation of IL8 expression and poor clinical outcome for breast cancer patients has been evident in the literature [77,78]; however, how IL8 contributes to poor outcome on the tumor cells has not been fully resolved. Here, we conclusively showed that IL8 is greatly overexpressed specifically in the migratory subpopulation of primary breast tumor cells and that its function is required for tumor cell invasion and hematogenous dissemination *in vivo*.

A significant novelty of the human invasion signature identified here is that it is specific to the early steps of the metastatic cascade, migration and invasion inside the primary tumor, two processes that are initiated by chemotactic cues in specific tumor microenvironments [7]. MDA-MB-231 cells have been used before to devise signatures specific to organ-tropic colonization to bone [13], to lung [14], and to brain [15], as well as a signature of circulating tumor cells (CTCs) self-seeding back to the primary tumor [79]. We also used MDA-MB-231 cells as our metastatic human breast cancer cell model, and we devised a signature that is specific to migration and invasion inside the primary tumor, a step of the metastatic cascade that precedes the metastatic steps analyzed in the previously mentioned studies. The Human Invasion Signature (HIS) derived in our study consists of a unique gene list that has little overlap with these previously MDA-MB-231-derived organ-tropic specific signatures. This agrees with a hypothesis of different gene-expression programs being crucial for each step of the metastatic cascade. In addition, a recent intravital imaging report by Giamperi *et al*. [80] showed activation of TGF-β signaling on migration of rat MTLn3 mammary tumor cells toward blood vessels in the primary tumor but subsequent downregulation of the same pathway for successful establishment of lung metastasis, again suggesting that each step of the metastatic cascade has different gene-expression programs. In the study presented here, we show that nearly all actively migrating tumor cells isolated from patient-derived human breast tumors have active TGF-β signaling, and that functional blocking of this signaling leads to significantly decreased invasion and hematogenous dissemination *in vivo*. Collectively, these data emphasize the need for high-resolution studies into defining the exact contributions of genes and signaling pathways in each tumor cell subpopulation and each step of tumor progression to have a complete picture of the timing of their expression and exact contribution to metastatic progression.

TGF-β signaling has been previously implicated in epithelial-to-mesenchymal transition (EMT), as well as maintenance of tumor-initiating cell (TIC) phenotypes [81,82]. Because we showed that TGF-β is a central regulator of the upregulated genes of our signature and also found that the migratory cells have active TGF-β signaling during invasion in the primary tumor *in vivo*, this raises the question that our signature may have some overlap with EMT or TIC gene-expression profiles. When we tested our signature for potential enrichment for an EMT signature, we indeed found a significant positive correlation of the EMT downregulated genes in the Taube *et al*. signature [83] with the downregulated genes in our HIS signature; however, no significant correlation for the upregulated genes was found in the two signatures (see Additional File 9). This could be because our signature is derived from MDA-MB-231 cells, which are already somewhat mesenchymal. As far as TIC signatures are concerned, GSEA comparison of the HIS with three published TIC signatures [36,84,85] showed a trend for anti-correlation between our signature and the tumor-initiating gene profile (that is, genes that are upregulated in TICs are significantly enriched in the downregulated genes of the HIS, whereas genes that are downregulated in TICs are significantly enriched in the upregulated genes of the HIS (see Additional File 9)). Interestingly, GSEA reported multiple signatures of normal embryonic stem cells [86-89] as being significantly enriched in the HIS (see Additional File 9). This evidence would suggest that migratory tumor cells at the particular moment of active migration while invading in the primary tumors acquire gene-expression profiles similar to cells during development, when migration is required for normal morphogenesis. It is possible that, at that particular moment, a gene-expression profile that contributes to tumor initiation (that is, growth) is switched off, as this capacity would be required only after the tumor cell has potentially arrived at its final destination of a metastatic target organ. Indeed, we recently showed that the growth and invasion capabilities of metastatic breast tumor cells *in vivo *can be uncoupled and oppositely regulated, with the nonreceptor kinase Arg/Abl2 acting as a switch to govern the cell decision to either "grow" or "go" [90].

One of the most novel and significant findings of our study is the importance of *IL8 *and *PTPN11 *in invasion and intravasation of human breast tumors. Blocking of the functions of these gene products significantly abrogated *in vivo *invasion and tumor cell dissemination in both MDA-MB-231 and patient-derived tumors, suggesting a significant role of these factors in the early steps of the metastatic cascade. Interestingly, PTPN11 and a receptor for IL8, CXCR1, have also been implicated in cancer stem cell self-renewal in the breast [59,84,91]. This dual role for these genes could potentially render them attractive targets for breast cancer therapy. Ginestier and colleagues [91] also showed that blocking of both the receptors for IL8, CXCR1, and CXCR2, by treatment with the drug repertaxin, significantly reduced the formation of bone metastasis after intracardiac injection of breast tumor cells in mice. However, this type of experimental metastasis assay artificially introduces the tumor cells in the bloodstream and completely skips the metastatic steps of invasion, migration, and intravasation in the primary tumor, so the decreased metastasis could be partially explained by the property of this drug to affect self-renewal. Here, we show a direct role for IL8 in primary tumor invasion and intravasation. A more-detailed study of the exact mechanism of the role of IL8 in invasion and intravasation in primary mammary tumors, and whether that uses the CXCR1 or CXCR2 receptors on the tumor cells or a paracrine interaction with the tumor stroma, is under way.

Finally, it has been argued that because dissemination from the primary tumor can occur early in cancer progression, potentially before clinical presentation [92], antiinvasion and antidissemination therapy may not be a plausible target for cancer therapy. However, many recent studies strongly point to invasion and dissemination as being clinically relevant targets after resection of the primary tumor: (a) tumor cells can disseminate from metastatic sites and seed back to the primary tumor site or other metastatic sites [79]; (b) CTCs can be found in the blood of patients years or decades after the removal of their primary tumor [93], suggesting that secondary deposits of tumor cells in the body of the patient can still invade and disseminate regularly into the blood circulation; and (c) the number of CTCs in the peripheral blood of patients is prognostic of cancer recurrence and poor survival [94-96], suggesting that these cells are causative of further metastasis. In the end, the main reason that therapeutics are not currently being developed to target for invasion and dissemination is the lack of relevant therapeutic end points and appropriate trial design in current clinical practice. However, research effort is being put into changing these ideas. Including information about expression patterns that are specific to the steps of intravasation and dissemination would provide valuable insights into pathways with potential importance for dissemination and inhibitors of them. With more research shedding light on the specific steps of invasion, dissemination, and metastasis, such development of novel end points, prognostics, and potentially, therapeutics may be feasible in clinical practice in the future.

## Conclusions

We have explored the gene-expression profile of the specific subpopulation of primary breast tumor cells captured while undergoing invasion inside the primary tumor *in vivo*. We therefore identified a gene signature specific to the early metastatic steps of migration and invasion inside the primary tumor. Our study proposes a new approach to cancer-expression profiling, in which specific stages of metastatic progression are analyzed, to gain more-detailed and temporally specific information. Such high-resolution knowledge about the genetic events that drive individual steps of metastasis will be imperative for a more in-depth understanding of cancer progression, as well as for improved design of prognostic and therapeutic tools for breast cancer.

## Abbreviations

BrdU: bromodeoxyuridine; BSA: bovine serum albumin; CDC25A: cell-division cycle 25 homolog A; CDC42: cell division control protein 42; CSF1: colony-stimulating factor 1; CSF1R: colony-stimulating factor 1 receptor; CTC: circulating tumor cell; DAPI: 4',6-diamidino-2-phenylindole; DAZAP2: DAZ-associated protein 2; DMEM: Dulbecco modified Eagle medium; DMSO: dimethyl sulfoxide; EGF: epidermal growth factor; ER: estrogen receptor; FBS: fetal bovine serum; FDR: false discovery rate; GAPDH: glyceraldehyde 3-phosphate dehydrogenase; GFP: green fluorescent protein; GSEA: gene-set enrichment analysis; HIS: human invasion signature; HT: human tumor; IHC: immunohistochemistry; IL8: interleukin 8; H&E: hematoxylin and eosin; IPA: ingenuity pathway analysis; KEGG: Kyoto Encyclopedia of Genes and Genomes; KLF11: Kruppel-like factor 11; NCL: nucleolin; NPM1: nucleophosmin; PBS: phosphate-buffered saline; PCR: polymerase chain reaction; PTPN11: protein tyrosine phosphatase: nonreceptor type 11; RT-PCR: reverse-transcription polymerase chain reaction; SAM: significance analysis of microarrays; SCID: severe combined immunodeficiency; TAM: tumor-associated macrophage; TGF-β: transforming growth factor-beta; VAMP7: vesicle-associated membrane protein 7.

## Competing interests

JC and AP have filed a patent application on the contents of this article. YW, JL, KW, SG, and PAK declare that they have no competing interests.

## Authors' contributions

AP and JC conceived of and designed the overall study. AP designed, performed, and interpreted the microarray analysis, the IPA and GSEA analysis, the real-time PCR analysis, and the mouse validation experiments, and wrote the manuscript. YW performed and interpreted mouse experiments and edited the manuscript. JL performed biostatistics analysis and edited the manuscript. KW supervised the patient tissue procurement, performed the histopathology characterization of the human tumor samples, and edited the manuscript. SG contributed in the design and interpretation of the microarray experiments. PAK performed the signature-validation analyses in previously published datasets and edited the manuscript. JC designed and interpreted experiments, and co-authored the manuscript. All authors read and approved the manuscript for publication.

## Supplementary Material

Additional File 1 **Schematic and additional discussion of experimental methods and technical controls for the microarray analysis**. Click here for file

Additional File 2 **Gene list for the human invasion signature**. Contains the complete list of genes upregulated and downregulated in the HIS, together with notes on gene functions and annotations. Also contains the smaller gene list of the highest regulated genes that was used in the Cox-proportional hazard regression modeling analyses. Click here for file

Additional File 3 **Table of sequences for primers used in real-time RT-PCR analysis of **Figure 2. Click here for file

Additional File 4 **Regulatory network map for HIS-upregulated genes involved in the functional network "DNA Replication and Repair."**. Click here for file

Additional File 5 **Regulatory network map for HIS-upregulated genes involved in the functional networks Embryonic and tissue development and cellular movement and development**. Click here for file

Additional File 6 **Results from IPA and GSEA canonic pathway analysis of the HIS**. Click here for file

Additional File 7 **Characterization of the patient-derived xenograft tumors**. Contains detailed tables explaining for each patient-derived xenograft: **(A) **the pathologic characteristics of the original patient tumor; **(B) **the growth, invasion, and metastasis properties of the xenograft tumors as grown in mice. Click here for file

Additional File 8 **Functional control for *Myc *inhibition *in vivo*. **Injection of the MYC inhibitor 10058-F4 in MDA-MB-231 xenograft mice significantly inhibits proliferation *in vivo*, as shown by reduced BrdU incorporation in the primary tumor. Click here for file

Additional File 9 **Results from Gene-Set Enrichment Analysis (GSEA) analysis of the HIS toward published signatures**. Click here for file
