# Supplementary material for: Selective gene-expression profiling of migratory tumor cells in vivo predicts clinical outcome in breast cancer patients
Source: Breast Cancer Res. 2012 Oct 31;14(5):R139. doi: 10.1186/bcr3344 (PMC4053118; doi:10.1186/bcr3344)
Supplement: Additional File 1 — Schematic and additional discussion of experimental methods and technical controls for the microarray analysis. [file bcr3344-S1.PDF]

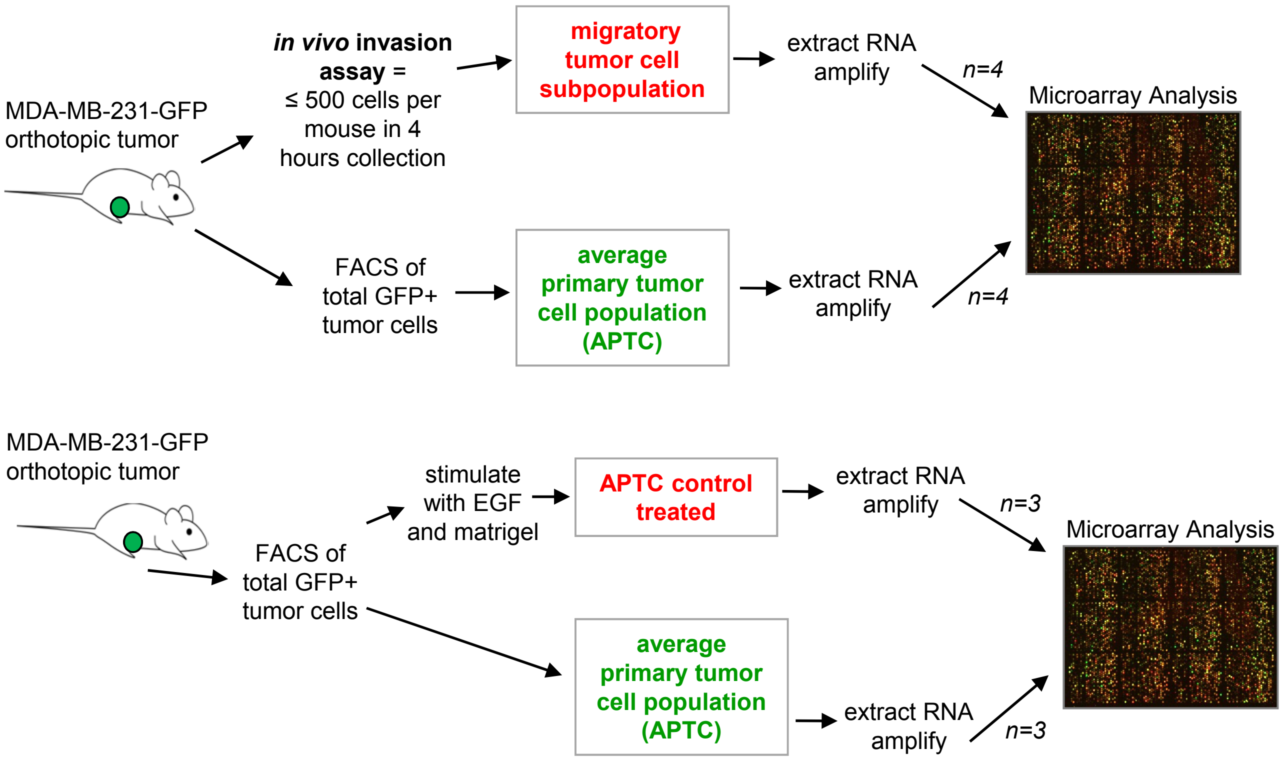

Additional File 1: Deriving the human invasion signature.

Schematic of the experimental method for the gene expression analysis of invasive human breast tumor cells. Orthotopic xenografts of human MDA-MB-231-GFP breast adenocarcinoma cells were made in SCID mice. Migratory cells were isolated with the *in vivo* invasion assay, where cells are stimulated to migrate towards an EGF gradient. The average primary tumor cells (APTCs) were isolated by FACS sorting for live GFP-positive cells from a whole tumor cell preparation. Both populations are tumor cells by more than 95% purity: we have shown that invasive cells from MDA-MB-231 tumors consist 95% tumor cells (Patsialou *et al*., 2009), and the purity of the APTCs was determined by post-sort FACS analysis. RNA was extracted from both the purified cell populations. Because the *in vivo* invasion assay results in 300-500 tumor cells per 4 hours collection per mouse, amplification of total cDNA is performed in all samples prior to microarray hybridization. A total of 4 biological repeats was used per sample for the analysis (discussed further in Methods).

In addition, we controlled for the conditions of cell collection to ensure that the invasion gene signature for the tumor cells is independent of the cell collection method. We therefore used three additional biological repeats of APTCs that we treated with matrigel and EGF inside needles *ex vivo*, to mimic the conditions of the *in vivo* invasion assay. These control samples were used for RNA extraction, amplification, hybridization and quantification in exactly the same method as the experimental samples. Statistical analysis of the control samples versus the APTCs gave a list of genes upregulated solely due to the matrigel/EGF stimulation. These genes were subtracted from our final signature, so that the Human Invasion Signature would account for the gene profile of the breast tumor cells while they migrate and invade *in vivo* through the tumor microenvironment, and not the cell collection method.

Additional controls performed in previous studies for this assay, to exclude that the migration measured in this assay is not a result of local inflammation because of the insertion of the microneedles:

a. *in vivo* invasion assay in normal mammary tissue showed no significant migration to the gradient
b. *in vivo* invasion assay in mammary primary tumors in the absence of a chemotactic gradient (only matrigel in the microneedles) shows no significant migration.
