# Supplementary material for: Selective gene-expression profiling of migratory tumor cells in vivo predicts clinical outcome in breast cancer patients
Source: Breast Cancer Res. 2012 Oct 31;14(5):R139. doi: 10.1186/bcr3344 (PMC4053118; doi:10.1186/bcr3344)
Supplement: Additional File 3 — Table of sequences for primers used in real-time RT-PCR analysis of Figure 2. [file bcr3344-S3.PDF]

GENE	Forward primer	Reverse primer
GAPDH	cgaccactttgtcaagctca	ccctgttgctgtagccaaat
B2M	gctcgcgctactctctcttt	ttcaatgtcggatggatgaa
ARHGDIB	ctcggcctgaggagtatgag	gtggtcttgcttgtcatcgt
CAPZA2	tacgtcgacagttgccagtt	tctgcatctctttgccaatc
CAPZB	atatcgtcaatgggctgagg	ctcttcaaagcctccaccag
SNTB2	ctgctgagctgatcaaggaa	cggtacaatatgctgctgga
PHACTR2	agaggcccacaactgaagaa	ggctgagctttctgctgagt
TUBA1A	ccaagcgtaccatccagttt	agtgggaggctggtagttga
CAV1	cgtctgtgacccactctttg	gatgcggacattgctgaata
CDC42	tacgaccgctgagttatcca	atctcaggcacccacttttc
IL8	ctgcgccaacacagaaatta	acttctccacaaccctctgc
LSM3	gacgacgtagaccagcaaca	cgaagctctcggtcatttct
MSN	aaggagagtgaggctgtgga	gctctgccacatgaggtgta
PTPN11	atatggcgtcatgcgtgtta	tccgtattcccttgtccaac
SMAD2	gtgcaatctttgtgcagagc	agcagcaaattcctggttgt
SNTB2	ctgctgagctgatcaaggaa	cggtacaatatgctgctgga
FADD	gacctccagaacaggagtgg	atgcgtctgagttccatgac
KLF11	gccggaagacctacttcaaa	gctgcagttgaaaggcttct
VAMP7	gctcgagccatgtgtatgaa	tccaccacagagaggtgaaa
YWHAE	gcagaactggatacgctgag	cctgcatgtctgaagtccat
DAZAP2	tggtggaaggagggtatgat	aggaggtggaggaggaatgt
FOXM1	tgatggatctcagcaccact	gggacggagatgaggtctaa
CDC25A	cccaaactccactaccctga	gcggaacttcttcaggtctt
CKS1B	atagccaagctggtccctaa	tgtgaggttctggttcatgg
IFI16	catggacgactgaccacaat	cctggtcttgatgaccttga
NCL	ttcaacagtgaggaggatgc	agccaccttcacccttaggt
NPM1	ggtggttctcttcccaaagt	agcctcttggtcagtcatcc
POLR2G	tgattcagcaggacgatgag	tcagcttacaagccccaagt
S100A11	tgccttcacaaagaaccaga	ccttgaggaaggagtcatgg
SKP1	accctcctcctcctgaagat	cttggtcccaaacagggata
TRIM32	tcgccagattagccacttct	tggagaatttccttgcgact
UBC	cgtgaagaccctgactggta	cttggatctttgccttgaca
XRCC5	cctgaaagcccttcaagaga	agaggcttcctctttggtga

<u>Additional File 3</u>:
Primer sequences for real-time PCR validation of Figure 2.
