# Supplementary material for: Selective gene-expression profiling of migratory tumor cells in vivo predicts clinical outcome in breast cancer patients
Source: Breast Cancer Res. 2012 Oct 31;14(5):R139. doi: 10.1186/bcr3344 (PMC4053118; doi:10.1186/bcr3344)
Supplement: Additional File 4 — Regulatory network map for HIS-upregulated genes involved in the functional network "DNA Replication and Repair.". [file bcr3344-S4.PDF]

Network 1: DNA Replication and Repair

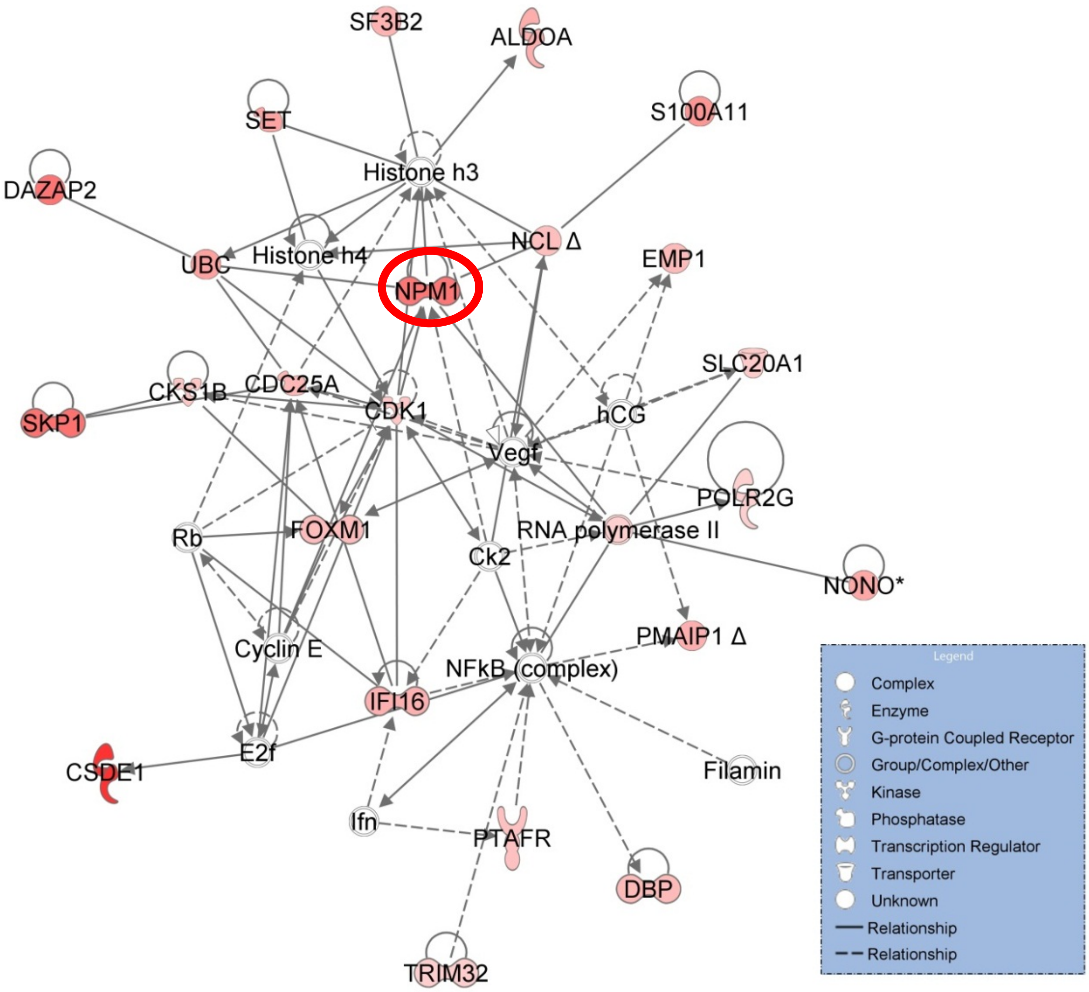

<u>Additional File 4:</u>

Map of protein interactions of Network 1.

Molecules in red shapes are upregulated in the Human Invasion Signature, molecules in clear shapes are inserted in the interaction map by the software to complete interaction links. Lines denote interactions between proteins, direct when the line is full or indirect when the line is dotted.

The red circle denotes the protein that was chosen as a target for the inhibition studies.
