# Supplementary material for: Selective gene-expression profiling of migratory tumor cells in vivo predicts clinical outcome in breast cancer patients
Source: Breast Cancer Res. 2012 Oct 31;14(5):R139. doi: 10.1186/bcr3344 (PMC4053118; doi:10.1186/bcr3344)
Supplement: Additional File 5 — Regulatory network map for HIS-upregulated genes involved in the functional networks Embryonic and tissue development and cellular movement and development. [file bcr3344-S5.PDF]

Network 2: Embryonic and Tissue Development
Network 3: Cellular Movement and Development

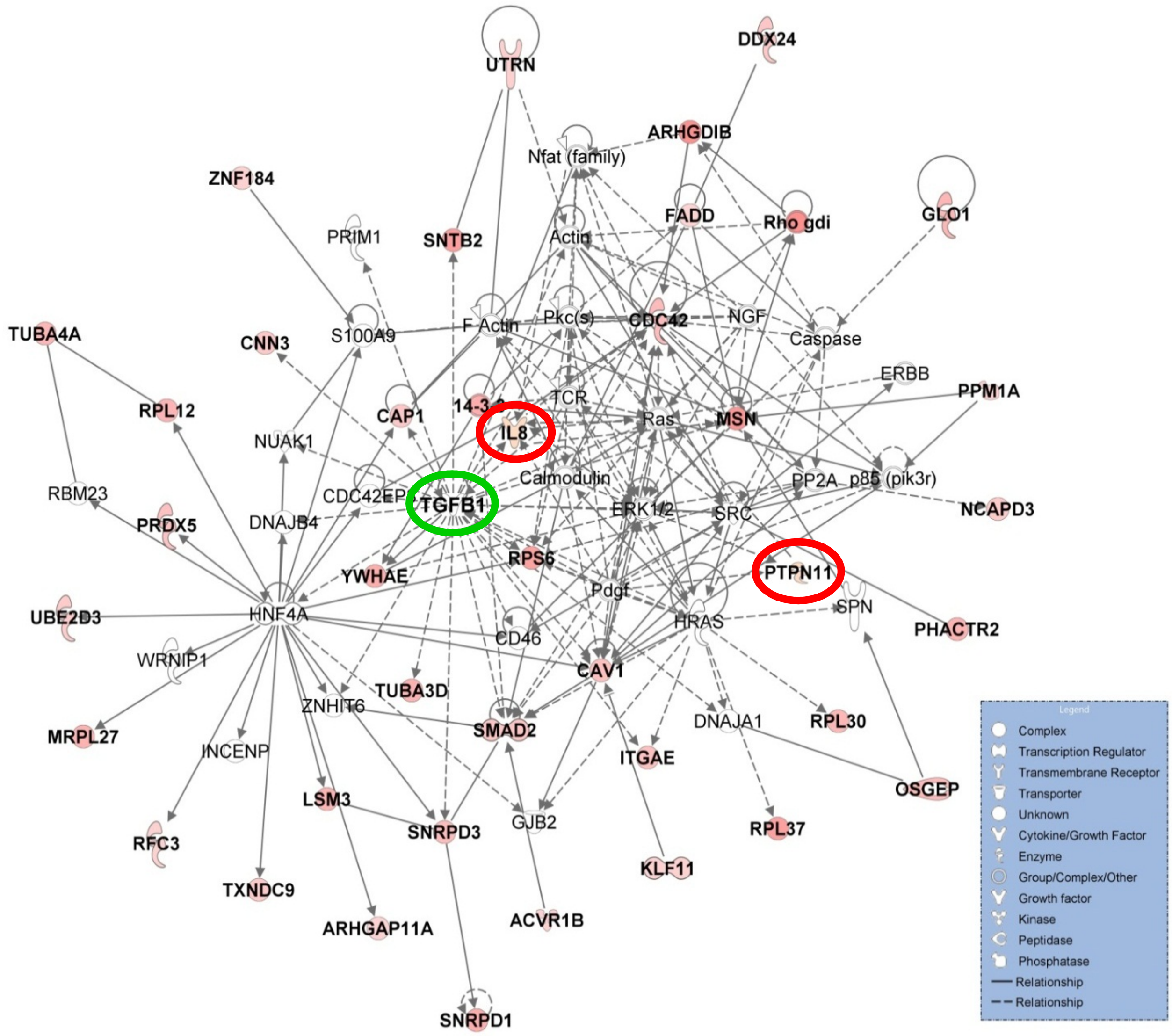

<u>Additional File 5:</u>

Map of protein interactions of Network 2 and 3.

Networks 2 and 3 had the most overlapping genes and therefore a common interaction map was generated. Molecules in red shapes are upregulated in the Human Invasion Signature, molecules in clear shapes are inserted in the interaction map by the software to complete interaction links. Lines denote interactions between proteins, direct when the line is full or indirect when the line is dotted.

The red circles denote the proteins that was chosen as targets for the inhibition studies. The green circle denotes TGFβ1, which although itself is not regulated, it is a central node of interaction for the HIS upregulated genes.
