# Supplementary material for: Selective gene-expression profiling of migratory tumor cells in vivo predicts clinical outcome in breast cancer patients
Source: Breast Cancer Res. 2012 Oct 31;14(5):R139. doi: 10.1186/bcr3344 (PMC4053118; doi:10.1186/bcr3344)
Supplement: Additional File 6 — Results from IPA and GSEA canonic pathway analysis of the HIS. [file bcr3344-S6.PDF]

A

Top regulated pathways	-log(p-value)
Oxidative Phosphorylation	3.27
CHK Proteins in Cell Cycle Checkpoint Control	2.86
Cell Cycle: G2/M DNA Damage Checkpoint Regulation	2.65
Purine Metabolism	2.31
Protein Ubiquitination Pathway	2.06
TGF-β Signaling	1.82
Integrin Signaling	1.4
ATM Signaling	1.34

B

KEGG Gene Set	ES	NES	Nominal p-value
HSA00190_OXIDATIVE_PHOSPHORYLATION	0.5359	1.6825	0.0000
HSA00240_PYRIMIDINE_METABOLISM	0.4525	1.5815	0.0000
HSA00230_PURINE_METABOLISM	0.3952	1.4703	0.0000
HSA03020_RNA_POLYMERASE	0.5640	1.4447	0.0000
HSA04350_TGF_BETA_SIGNALING_PATHWAY	0.3329	1.3473	0.0106
HSA00970_AMINOACYL_TRNA_BIOSYNTHESIS	0.5090	1.4737	0.0198
HSA00010_GLYCOLYSIS_AND_GLUCONEOGENESIS	0.5050	1.5838	0.0257
HSA03010_RIBOSOME	0.7261	1.6892	0.0264
HSA04115_P53_SIGNALING_PATHWAY	0.4093	1.4731	0.0308
HSA00620_PYRUVATE_METABOLISM	0.5118	1.4412	0.0357
HSA00252_ALANINE_AND_ASPARTATE_METABOLISM	0.4376	1.4437	0.0502
HSA04810_REGULATION_OF_ACTIN_CYTOSKELETON	0.3758	1.4935	0.0651
HSA00590_ARACHIDONIC_ACID_METABOLISM	0.4484	1.3874	0.0689
HSA04110_CELL_CYCLE	0.3951	1.4529	0.0938

Additional File 6:

Enriched canonical pathways in the Human Invasion Signature.

A. IPA analysis of the HIS towards canonical pathways. Shown are the pathways that were designated significantly enriched by the software, with a p-value <0.05 by Fisher's Exact test.

B. Gene Set Enrichment Analysis (GSEA) of the HIS towards the KEGG list of gene sets (available at the GSEA Molecular Signatures database). Shown are the pathways designated by the software as significant based on FDR<25%. ES: Enrichment Score, NES: Normalized Enrichment Score.
