# Supplementary material for: Selective gene-expression profiling of migratory tumor cells in vivo predicts clinical outcome in breast cancer patients
Source: Breast Cancer Res. 2012 Oct 31;14(5):R139. doi: 10.1186/bcr3344 (PMC4053118; doi:10.1186/bcr3344)
Supplement: Additional File 7 — Characterization of the patient-derived xenograft tumors. Contains detailed tables explaining for each patient-derived xenograft: (A) the pathologic characteristics of the original patient tumor; (B) the growth, invasion, and metastasis properties of the xenograft tumors as grown in mice. [file bcr3344-S7.PDF]

A

Sample ID	Pathological diagnosis	Grade	ER	PR	Her2	AJCC stage
**HT1**	Invasive ductal carcinoma	HG-9	Neg	Neg	Neg	T2N1micMx
**HT3**	Invasive lobular carcinoma, pleomorphic type	n/a	Pos	Pos	Neg	T1bN0Mx
**HT17**	Invasive ductal carcinoma	HG-9	Neg	Neg	Neg	T4bN0Mx
**HT24**	Invasive ductal carcinoma	HG-8	Pos	Pos	Neg	T1cN0(i+)Mx
**HT30**	Invasive ductal carcinoma	HG-9	Pos	<2%	Neg	T2N0Mx
**HT33**	Invasive ductal carcinoma	HG-9	Pos	Neg	Neg	T2N0Mx
**HT34**	Invasive ductal carcinoma	HG-9	Neg	Neg	Neg	T1cN0Mx
**HT39**	Invasive ductal carcinoma	HG-9	Neg	Neg	Neg	T4dNxMx

B

Sample ID	Latency (months)	Passage in mice	In vivo invasion to FBS/EGF	Spontaneous lung metastasis
**HT1**	9	yes	yes (+)	yes
**HT3**	4	no	n/a	n/a
**HT17**	2	yes	yes (+++)	yes
**HT24**	5	no	n/a	n/a
**HT30**	6	yes	yes (+)	yes
**HT33**	3	yes	no	no
**HT34**	4	no	n/a	n/a
**HT39**	3	yes	yes (+++)	yes

Additional File 7:
Establishing a panel of patient-derived primary breast tumors in mice.
A. Pathological characteristics of the patient tumors that successfully grew a tumor in mice in first passage.
B. Propagation and metastatic properties of the patient-derived xenografts.
Latency: Time from surgery until tumor reaches approximately 1cm in diameter (number of months).
Passage in mice: Whether the sample had the capacity to re-grow tumors in subsequent mice after growth in the first mouse implanted (yes/no).
Invasion: *In vivo* invasion assay in response to EGF (or FBS as a general chemotactic signal) and whether cells migrate significantly to chemotactic gradients over no gradient control (yes/no, if yes +:<150 cells per microneedle, ++: 150-300 cells per microneedle, +++: >300 cells per microneedle).
Lung metastasis: Measurement of spontaneous lung metastasis in the mice bearing orthotopic tumors, presence or absence (yes/ no).
n/a: not applicable, measurement was not possible or not significant, because tumor grew only in the one initial implanted mouse and did not successfully propagate to other mice.
