# Supplementary material for: Selective gene-expression profiling of migratory tumor cells in vivo predicts clinical outcome in breast cancer patients
Source: Breast Cancer Res. 2012 Oct 31;14(5):R139. doi: 10.1186/bcr3344 (PMC4053118; doi:10.1186/bcr3344)
Supplement: Additional File 8 — Functional control for Myc inhibition in vivo. Injection of the MYC inhibitor 10058-F4 in MDA-MB-231 xenograft mice significantly inhibits proliferation in vivo, as shown by reduced BrdU incorporation in the primary tumor. [file bcr3344-S8.PDF]

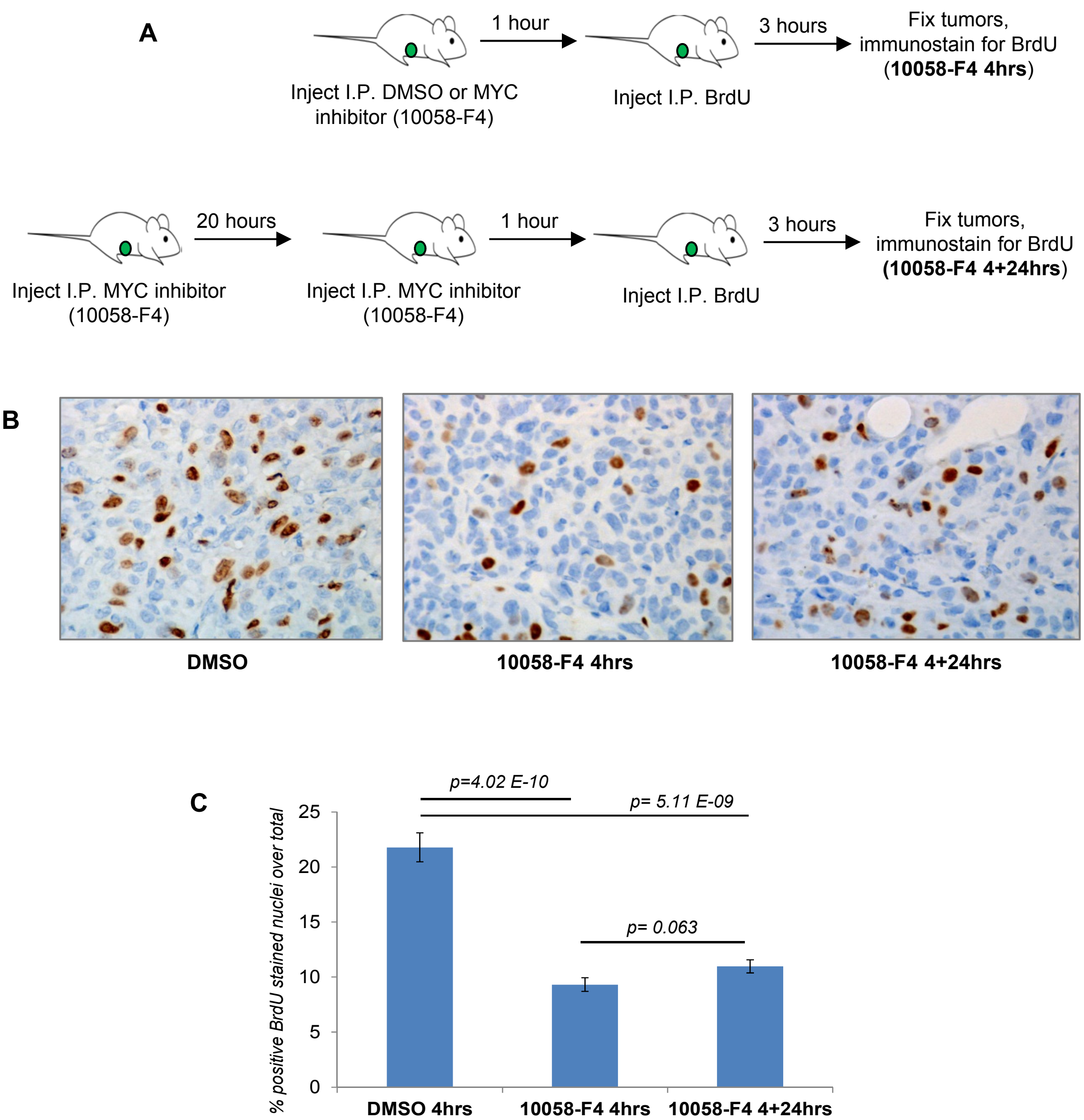

<u>**Additional file 8:**</u>

Injection of the MYC inhibitor 10058-F4 in MDA-MB-231 xenograft mice significantly inhibits proliferation *in vivo*.

A. Schematic of experimental design for the inhibitor treatment.

B. Representative images of immunostained tumors sections with BrdU antibody (brown) and counterstained for nuclei (blue).

C. Quantification of the above experiments is shown for 3 mice per group, and 10 random 40x images per mouse/tumor (excluding necrotic areas). Bars represent the average percentage of BrdU positive nuclei (brown) over total (blue). Error bars: SEM. p-values by Student's *t-test*.
