# Supplementary material for: Selective gene-expression profiling of migratory tumor cells in vivo predicts clinical outcome in breast cancer patients
Source: Breast Cancer Res. 2012 Oct 31;14(5):R139. doi: 10.1186/bcr3344 (PMC4053118; doi:10.1186/bcr3344)
Supplement: Additional File 9 — Results from Gene-Set Enrichment Analysis (GSEA) analysis of the HIS toward published signatures. [file bcr3344-S9.PDF]

A

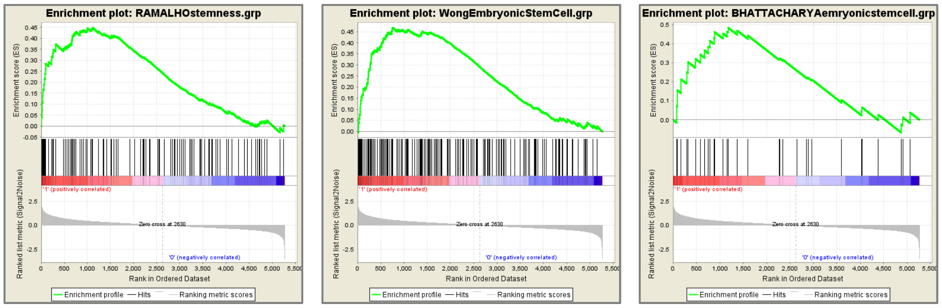

GENE SET	SIZE	ES	NES	NOM p-val	FDR q-val
**Ramalho_Stemness_Up**	**107**	**0.448**	**1.629**	**0.000**	**0.023**
**Wong_Embryonic_Stem_Cell_Core**	**169**	**0.468**	**1.643**	**0.000**	**0.032**
**Bhattacharya_Emryonic_Stem_Cell**	**31**	**0.483**	**1.436**	**0.039**	**0.082**
**BenPorath_ES_1**	**154**	**0.302**	**1.237**	**0.147**	**0.174**

B

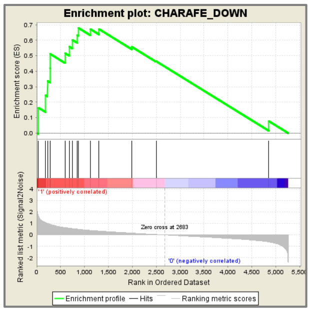

NAME	SIZE	ES	NES	NOM p-val	FDR q-val
**CHARAFE_DOWN**	**15**	**0.679**	**1.832**	**0.003**	**0.002**
CREIGHTON_DOWN	85	0.292	1.119	0.267	0.266
**CREIGHTON_UP**	**46**	**-0.3216**	**-1.2625**	**0.1329**	**0.1035**
LIU_DOWN	34	0.3809	1.2466	0.1727	0.3303
LIU_UP	35	0.3408	1.1114	0.3053	0.3034

C

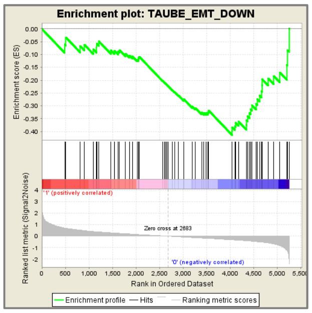

NAME	SIZE	ES	NES	NOM p-val	FDR q-val
TAUBE_EMT_UP	45	0.1978	0.6951	0.9247	0.9256
**TAUBE_EMT_DOWN**	**59**	**-0.4138**	**-1.6844**	**0.0031**	**0.0083**

D

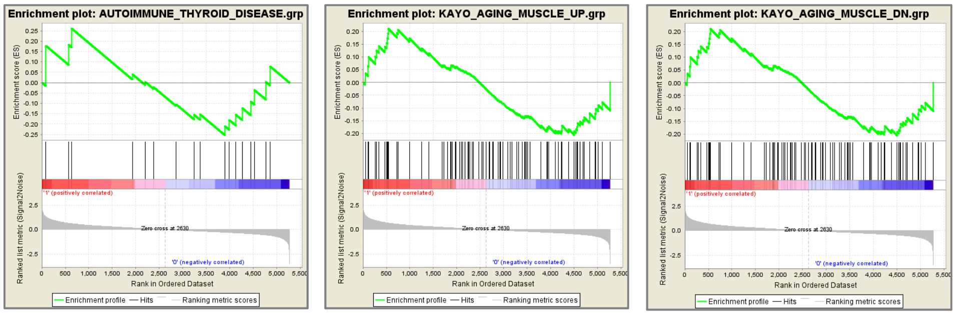

GENE SET	SIZE	ES	NES	NOM p-val	FDR q-val
Autoimmune_Thyroid_Disease	17	0.261	0.682	0.883	0.910
Kayo_Aging_Muscle_DN	83	0.211	0.856	0.675	0.707
Kayo_Aging_Muscle_UP	83	0.211	0.834	0.707	0.730

Additional file 9:

Gene Set Enrichment Analysis (GSEA) of the HIS towards published signatures.

Our microarray dataset was ranked from highly positive to highly negative differentially expressed genes between the Invasive tumor cells and the Average primary tumor cells (from red to blue respectively). Then GSEA evaluated the positions of the gene sets in question against this ranked list. A positive Enrichment Score (ES) denotes that the genes in the probed gene set are located as a group near the top of the ranked list and therefore more significant correlated with an upregulated phenotype. A negative ES denotes a correlation with a downregulated phenotype. As per the GSEA website, we considered significant gene sets with a False Discovery Rate (FDR) <25% (highlighted in bold through the figure).

A. GSEA toward curated datasets available at the Molecular Signature Database (MsigDB v3.0) (http://www.broadinstitute.org/gsea/msigdb/index.jsp) showed significant enrichment in the HIS for signatures related to embryonic stem cells. Signatures are listed in order of significance.

B. GSEA toward published Tumor-Initiating Cells (TIC) signatures. The list of upregulated genes in the Charafe-Jauffret et al. signature was not ranked by GSEA due to small list size and no overlap within the HIS. Overall, for the Charafe-Jauffret and the Creighton signatures, evidence for an inverse correlation between the TIC phenotype and the HIS was observed. Comparison to the Liu signature showed no significant enrichment with neither the upregulated nor the downregulated genes.

C. GSEA toward the published EMT core transciption signature by Taube et al. The EMT upregulated genes were not significantly enriched in the HIS. However, a significant correlation was observed between the EMT downregulated genes and the HIS downregulated genes (FDR<1%). Other EMT gene sets from the MsigDB Database were not ranked by GSEA to the HIS due to small list size.

D. As a negative control, random signatures of functions/phenotypes unrelated to cancer were also analyzed in parallel. No significant enrichment was observed (FDR > 70%).

References:

- Ramalho-Santos M, Yoon S, Matsuzaki Y, Mulligan RC, Melton DA: **"Stemness": transcriptional profiling of embryonic and adult stem cells**. *Science* 2002, **298**(5593):597-600.
- Wong DJ, Liu H, Ridky TW, Cassarino D, Segal E, Chang HY: **Module map of stem cell genes guides creation of epithelial cancer stem cells**. *Cell Stem Cell* 2008, **2**(4):333-344.
- Bhattacharya B, Miura T, Brandenberger R, Mejido J, Luo Y, Yang AX, Joshi BH, Ginis I, Thies RS, Amit M *et al*: **Gene expression in human embryonic stem cell lines: unique molecular signature**. *Blood* 2004, **103**(8):2956-2964.
- Ben-Porath I, Thomson MW, Carey VJ, Ge R, Bell GW, Regev A, Weinberg RA: **An embryonic stem cell-like gene expression signature in poorly differentiated aggressive human tumors**. *Nat Genet* 2008, **40**(5):499-507.
- Charafe-Jauffret E, Ginestier C, Iovino F, Wicinski J, Cervera N, Finetti P, Hur MH, Diebel ME, Monville F, Dutcher J *et al*: **Breast cancer cell lines contain functional cancer stem cells with metastatic capacity and a distinct molecular signature**. *Cancer Res* 2009, **69**(4):1302-1313.
- Creighton CJ, Li X, Landis M, Dixon JM, Neumeister VM, Sjolund A, Rimm DL, Wong H, Rodriguez A, Herschkowitz JI *et al*: **Residual breast cancers after conventional therapy display mesenchymal as well as tumor-initiating features**. *Proc Natl Acad Sci U S A* 2009, **106**(33):13820-13825.
- Liu R, Wang X, Chen GY, Dalerba P, Gurney A, Hoey T, Sherlock G, Lewicki J, Shedden K, Clarke MF: **The prognostic role of a gene signature from tumorigenic breast-cancer cells**. *N Engl J Med* 2007, **356**(3):217-226.
- Taube JH, Herschkowitz JI, Komurov K, Zhou AY, Gupta S, Yang J, Hartwell K, Onder TT, Gupta PB, Evans KW *et al*: **Core epithelial-to-mesenchymal transition interactome gene-expression signature is associated with claudin-low and metaplastic breast cancer subtypes**. *Proc Natl Acad Sci U S A* 2010, **107**(35):15449-15454.
